# Anchoring plant metallothioneins to the inner face of the plasma membrane of *Saccharomyces cerevisiae* cells leads to heavy metal accumulation

**DOI:** 10.1371/journal.pone.0178393

**Published:** 2017-05-31

**Authors:** Lavinia Liliana Ruta, Ya-Fen Lin, Ralph Kissen, Ioana Nicolau, Aurora Daniela Neagoe, Simona Ghenea, Atle M. Bones, Ileana Cornelia Farcasanu

**Affiliations:** 1 Faculty of Chemistry, University of Bucharest, Bucharest, Romania; 2 Cell, Molecular Biology and Genomics Group, Department of Biology, Norwegian University of Science and Technology, Trondheim, Norway; 3 Faculty of Biology, University of Bucharest, Bucharest, Romania; 4 Institute of Biochemistry of the Romanian Academy, Bucharest, Romania; Texas A&M University, UNITED STATES

## Abstract

In this study we engineered yeast cells armed for heavy metal accumulation by targeting plant metallothioneins to the inner face of the yeast plasma membrane. Metallothioneins (MTs) are cysteine-rich proteins involved in the buffering of excess metal ions, especially Cu(I), Zn(II) or Cd(II). The cDNAs of seven *Arabidopsis thaliana* MTs (*At*MT1a, *At*MT1c, *At*MT2a, *At*MT2b, *At*MT3, *At*MT4a and *At*MT4b) and four *Noccaea caerulescens* MTs (*Nc*MT1, *Nc*MT2a, *Nc*MT2b and *Nc*MT3) were each translationally fused to the C-terminus of a myristoylation green fluorescent protein variant (*myr*GFP) and expressed in *Saccharomyces cerevisiae* cells. The *myr*GFP cassette introduced a yeast myristoylation sequence which allowed directional targeting to the cytosolic face of the plasma membrane along with direct monitoring of the intracellular localization of the recombinant protein by fluorescence microscopy. The yeast strains expressing plant MTs were investigated against an array of heavy metals in order to identify strains which exhibit the (hyper)accumulation phenotype without developing toxicity symptoms. Among the transgenic strains which could accumulate Cu(II), Zn(II) or Cd(II), but also non-canonical metal ions, such as Co(II), Mn(II) or Ni(II), *myr*GFP-*Nc*MT3 qualified as the best candidate for bioremediation applications, thanks to the robust growth accompanied by significant accumulative capacity.

## Introduction

Heavy metals such as Cu(II), Mn(II), Ni(II), Co(II) and Zn(II) are of unique importance for life, being included in the shortlist of trace elements which are essential for the activity of various biomolecules, particularly enzymes. These elements are taken up by organisms from their environment through a variety of high or low affinity transport systems, which constantly provide the cell the required amount of essential metals. Metal composition of the environment can fluctuate due to a series of natural or anthropogenic activities, preponderantly related to industry. Drastic changes in heavy metal normal occurrence can be detrimental to normal life, leading to toxic effects (heavy metal pollution) or deficiencies (essential heavy metal scarceness) [1, 2].

Heavy metal pollution represents a threat to living organisms as the excess metals (both essential and non-essential) attack non-specifically the innate uptake systems, penetrating the cells in high concentration, where they exert their deleterious effects by non-specifically binding to any molecule that would bear a negative charge. The removal of excess metals from contaminated sites is of utmost importance, but this has proved rather difficult, since heavy metals are natural components of the environment and are not degradable. Paradoxically, one of the most promising approaches for removal of the polluting metals is bioremediation, which makes use of organisms (natural or engineered) capable of accumulating large amounts of metals from their surroundings [3–6]. Among microorganisms, the yeast *Saccharomyces cerevisiae* is a serious candidate for a position of heavy metal bioremediator. Apart from being a GRAS (generally regarded as safe) microorganism, *S*. *cerevisiae* has an innate high biosorptive capacity due to the chemical structure of the cell wall [7–9], which can be improved by yeast surface display techniques [10–17] or by manipulation towards obtaining heavy metal accumulating phenotypes [18, 19]. Naturally, *S*. *cerevisiae* is a non-accumulator, thanks to very active defense mechanisms used to limit the amount of metal ions within the living cells: in particular, excretion of excess metal ions via the secretory pathway is responsible for most of the heavy metal export [20, 21]. For bioremediation purposes, metal ions which enter the cells should be prevented from being excreted; this can be achieved by means of chemical ligands, which sequester the ions and also diminish their toxicity. Considering this possibility of metal export prevention, we attempted to obtain heavy metal accumulating yeast strains by arming the cells with plant metallothioneins (MTs) anchored to the inner face of the yeast plasma membrane.

MTs are metal-binding proteins found in all organisms [22]. These low-molecular mass proteins are cysteine-rich, and as a result they naturally bind to Cu(I), Zn(II) and Cd(II), having a protective role against metal toxicity achieved through the formation of sulfur-based metal-thiolate clusters [23]. Plant MTs are grouped into four subfamilies (MT1-MT4) based on sequence similarities, phylogenetic relationships and metal-binding domains [24, 25]. In yeast, the major Cu-activated MT Cup1 binds and sequesters Cu(I), providing the principal way of buffering this extremely toxic ion [26]. In the environment copper mainly exists as the more stable cupric ion, Cu(II), which is converted to the cuprous form Cu(I) by Fe/Cu reductases, to be further transported into the cell by Cu(I) transporters. Alternatively, Cu(II) is reduced in the cytosol by the reductive cell milieu. Due to its high reactivity Cu(I) is not allowed to exist freely in the cytosol, being buffered by efficient complexing agents, including MTs [27]. In the present study, copper will be specified as Cu(I) only when referred to thioneins; otherwise it will be presented as the more stable Cu(II). Although structurally dissimilar to yeast Cup1, MTs from the heavy metal non-hyperaccumulator *Arabidopsis thaliana* or from the hyperaccumulator *Noccaea caerulescens* were shown to functionally complement yeast *CUP1* mutations [28–31] indicating that MTs from these plant species bind metals when expressed in yeast. In previous attempts to increase the heavy metal bisorptive capacity for biotechnology purposes, yeast Cup1 variants were expressed at the surface of yeast cells by means of the yeast surface display technique [13, 14, 32]. In the afore mentioned studies it was revealed that yeast cells expressing on the cell surface either Cup1 fused with a hexahistidyl tag [13] or as tandem head-to-tail Cup1 repeats [14] had improved biosorption activity towards Cd(II). In a later study, engineered cell surface display yeasts expressing four types of *Solanum nigrum* MTs were shown to develop both Cd(II) tolerance and increased Cd(II) adsorption, exhibiting higher affinity for Cd(II) than for Cu(II) or Hg(II), along with a remarkable capacity to concentrate ultra-traces of Cd(II) at the cell surface [32].

In the present study, we addressed the possibility to obtain heavy metal hyperaccumulating *S*. *cerevisiae* by engineering cells towards producing plant MTs targeted to the inner face of the yeast plasma membrane. We hypothesized that the engineered yeast cells would accumulate heavy metals thanks to cation sequestration by the MTs attached to the cytosolic face of the membrane. The accumulative capacity of the engineered yeasts was tested under two conditions: (1) physiological, when “traces” of Co(II), Cu(II), Mn(II), Ni(II), Zn(II) and the non-essential Cd(II) were simultaneously present in the incubation medium, or (2) “tolerable excess”, when growth media were supplemented with individual metal ions introduced at the highest concentration that did not significantly affect cell viability. Under both conditions we identified strains which could accumulate Cu(II), Zn(II) or Cd(II), but also the MT-noncannonical Co(II), Mn(II) or Ni(II).

## Materials and methods

### Cloning plant MT cDNAs

Total RNA was extracted from the *A*. *thaliana* accession Col-0 and the *N*. *caerulescens* accession La Calamine with the Spectrum Plant Total RNA kit (Sigma-Aldrich, Saint Louis, USA) as described by the supplier. An on-column DNase digestion was performed using the RNase-Free DNase Set (Qiagen, Hilden, Germany) to eliminate genomic DNA. Total RNA was quantified using a NanoDrop ND-1000 (Nanodrop, Delaware, USA) and 1 μg total RNA was used to synthesize cDNA with the QuantiTect Reverse Transcription Kit (Qiagen, Hilden, Germany). The full-length coding sequence (minus the codon for the initial methionine) of seven *A*. *thaliana* and four *N*. *caerulescens* MTs was amplified from the respective cDNA with TaKaRa Ex Taq (Takara Bio Inc, Otsu, Japan) polymerase using the primers listed in S1 Table. The amplified products were purified from agarose gel using the Wizard SV Gel and PCR Clean-Up System (Promega Corporation, Madison, USA), cloned into the pCRII-TOPO vector by TOPO TA cloning (Life Technologies, Carlsbad, USA) and verified by sequencing (S1 Table). All cloned MT sequences were identical with those reported in NCBI, with the exception of *Nc*MT1, which contained a 24bp *in-frame* insert generating an additional NCGCGSSC close to the N-terminus and a single nucleotide polymorphism leading to a W to C change. These sequence features also exist in *At*MT1a and *At*MT1c. The sequence cloned for *Nc*MT3 contained two nucleotide polymorphisms leading to a Q to S change at position 18 when comparing to the *Nc*MT3 sequence published in NCBI. For each of the *At*MT4s different protein sequences are reported in NCBI; only one of each was cloned in the present study. The amino acid sequence alignment of the cloned MTs is presented in S1 Fig.

The MT-encoding inserts were excised from pCRII-TOPO using the restriction sites introduced by the primers during PCR amplification (S1 Table) and inserted into the same restriction sites of the yeast plasmid pGREG596 (*GAL1*::*myrGFP*, *URA3*, *HIS3*) [33], purchased from EUROSCARF (European *S*. *cerevisiae* Archive for Functional Analysis, www.euroscarf.de). This directional cloning allowed the removal of the *HIS3* gene from pGREG596 and introduced the plant *MT* sequences *in frame* downstream of the *myrGFP* gene (S2 Table). The resulting yeast plasmids harboring plant MT ORFs (minus initial methionine) are presented in Table 1.

**Table 1:** Plasmids used to clone and express *myr*GFP-MTx.

Metallothionein harbored	pCRII—MTx pCRII-based plasmid (obtained by TA cloning)	pGRD-myrGFP::MTx pGREG596-based plasmid (*GAL1* promoter, *URA3*)	Chimeric protein
**Control**	pCRII-myrGFP	pGRD-myrGFP	*myr*GFP
***Sc*MT (*CUP1*)**	pCRII—CUP1	pGRD-myrGFP::CUP1	*myr*GFP-Cup1
***At*MT1a**	pCRII-AtMT1a	pGRD-myrGFP:: AtMT1a	*myr*GFP- *At*MT1a
***At*MT1c**	pCRII- AtMT1c	pGRD-myrGFP:: AtMT1c	*myr*GFP- *At*MT1c
***At*MT2a**	pCRII- AtMT2a	pGRD-myrGFP:: AtMT2a	*myr*GFP- *At*MT2a
***At*MT2b**	pCRII- AtMT2b	pGRD-myrGFP:: AtMT2b	*myr*GFP- *At*MT2b
***At*MT3**	pCRII- AtMT3	pGRD-myrGFP:: AtMT3	*myr*GFP- *At*MT3
***At*MT4a**	pCRII- AtMT4a	pGRD-myrGFP:: AtMT4a	*myr*GFP- *At*MT4a
***At*MT4b**	pCRII- AtMT4b	pGRD-myrGFP:: AtMT4b	*myr*GFP- *At*MT4b
***Nc*MT1**	pCRII- NcMT1	pGRD-myrGFP:: NcMT1	*myr*GFP- *Nc*MT1
***Nc*MT2a**	pCRII- NcMT2a	pGRD-myrGFP:: NcMT2a	*myr*GFP- *Nc*MT2a
***Nc*MT2b**	pCRII- NcMT2b	pGRD-myrGFP:: NcMT2b	*myr*GFP- *Nc*MT2b
***Nc*MT3**	pCRII- NcMT3	pGRD-myrGFP:: NcMT3	*myr*GFP- *Nc*MT3

The primers used to amplify metallothioneins (MTs) from *Arabidopsis thaliana* (*At*MTs) or *Noccaea caerulescens* (*Nc*MTs) cDNA are listed in S1 Table and were designed to fuse *myr*GFP with the corresponding MT lacking the first methionine. The MT gene from *Saccharomyces cerevisiae*, *CUP1* is intronless and it was amplified using genomic DNA as template. To amplify the *myr*GFP control, pGREG596 (http://web.uni-frankfurt.de/fb15/mikro/euroscarf/data/pGREG.html) was used as template.

Yeast cells were transformed by the “quick and dirty” method [34] and the transformants were selected on SD-Ura agar plates. Correct transformation was double-checked by colony PCR [33].

### Yeast strains and culture conditions

The *S*. *cerevisiae* strain used in this study was BY4741 (*MAT*a; *his3Δ1*; *leu2Δ0*; *met15Δ0*; *ura3Δ0*), obtained from EUROSCARF. Cell storage, growth media and cell manipulation were done as described [35]. Transformed strains were maintained and grown in standard SD-Ura (synthetic media with dextrose lacking uracil, for plasmid selection) or SGal-Ura (synthetic media with galactose, for transgene induction). Minimal defined media (MMe) were virtually metal free and were supplemented with the essential elements and with controlled metal concentrations prior to use. For solid media, 2% agar was used. For growth improvement, all the synthetic media were supplemented with 40 mg/L leucine [36].

### Yeast cell growth assays

Transformants were grown overnight in SD-Ura and inoculated in fresh SD-Ura to OD_600_ = 0.05, then grown with agitation (30°C, 200 rpm) to OD_600_ = 0.1, which corresponded to approximately 10^6^ cells/mL. At this point (considered time 0) cells were harvested, washed and shifted to media containing galactose (SGal-Ura) for transgene induction. The cell viability was checked by staining with methylene blue and only populations with viability > 95% were used further. Cell growth in liquid media was determined at the specified times by determining OD_600_ in a plate reader equipped with thermostat and shaker (Varioskan, Thermo Fisher Scientific, Vantaa, Finland). To assess the effect of metal surplus on the growth or on the viability of cells expressing MTx, the cell suspensions were treated with MeCl_2_, added from sterile stocks 4 hours after the galactose shift. For growth on solid media, the cells prepared as above were 10-fold serially-diluted in a 48-well microtiter plate and stamped on agar plates using a pin replicator (approximately 4 μL/spot). Plates were photographed after incubation at 30°C for 3–6 days. The cell viability, expressed as percentage of live cells within a whole population, was assessed by staining with methylene blue. Viable cells were colorless, dead cells were blue. Viability was examined for at least 300 cells from one biological replicate.

### Metal accumulation by growing cells

Overnight pre-cultures were prepared as described above. At time 0, cells were shifted to SGal-Ura and incubated 4 h for transgene induction (30°C, 200 rpm). From this point, cells were used in two separate ways: (1) To determine metal accumulation under low-metal conditions, cells were washed and shifted to MMeGal-Ura (containing 2 μM MnCl_2_ and ZnCl_2_; 1 μM CuCl_2_, CoCl_2_, NiCl_2_ and CdCl_2_) and incubated for 10 h (30°C, 200 rpm) before being harvested for metal assay. (2) To determine metal accumulation under high concentration conditions, metal ions were added from sterile stocks to the indicated concentrations and incubated for 2 h (30°C, 200 rpm). To measure the metal accumulated, cells were harvested by centrifugation (1 min, 5000 rpm, 4°C) and washed three times with ice-cold 10 mM 2-(N-morpholino)ethanesulfonic acid (MES)-Tris buffer, pH 6.0. Cells were finally suspended in deionized water (10^8^ cells/mL). For metal assay, cells were digested with 65% ultrapure HNO_3_ (Merck, Darmstadt, Germany). Metal analysis was done using an instrument with a single collector, quadrupole inductively coupled plasma with mass spectrometry (ICP-MS, Perkin-Elmer ELAN DRC-e, Concord, Canada) against Multielement ICP Calibration Standard 3, matrix 5% HNO_3_ (Perkin Elmer Pure Plus). The metal cellular content was normalized to total cellular proteins, assayed spectrophotometrically as described [37]. Values were expressed as the mean ± standard deviation of triplicate determinations (technical triplicates) of three independent yeast transformants (biological replicates).

### *myr*GFP-MT localization

For the detection of *myr*GFP-MT fluorescence, the transformed cells were grown overnight in SD-Ura and manipulated as described above. At time 0, cells were shifted to SGal-Ura for transgene induction and grown for 2–4 hours before being visualized by fluorescence microscopy. Live cells were examined with an Olympus fluorescent microscope system (Olympus BX53, Tokyo, Japan) equipped with a HBO-100 mercury lamp and an Olympus DP73 camera. To detect the GFP signals, a GFP filter set (excitation filter 460–480, dichromatic mirror 585, emission filter 495–540) was used. The microscopic photographs were processed using the CellSens Dimension V1 imaging software (Olympus, Tokyo, Japan). For each strain, one representative image is shown.

### Isolation of mRNA and reverse transcription-PCR (RT-PCR)

Yeast transformants grown as described above were shifted to SGal-Ura for transgene induction. Four hours after the galactose shift, cells were harvested for RNA isolation. RNA was isolated using the RiboPure^™^ RNA Purification Kit for yeast (Ambion^™^, Thermo Fisher Scientific) following the manufacturer’s instructions. Approximately 50 ng RNA were used for each RT-PCR, using AccessQuick^™^ RT-PCR System (Promega Corporation, Madison, USA). The primers used to detect the expression of the transgene were presented in S3 Table. For each transformant, the expression of actin gene (*ACT1*) was used as control. The RT-PCR cycling conditions were 45°C, 30 min (for reverse transcription) followed by PCR: 95°C for 2 min, and 25 cycles of 95°C for 30 s, 55°C for 30 s, 68°C for 48 s.

### Reproducibility of the results and statistics

All experiments were repeated, independently, on three different transformants. For each individual experiment values were expressed as the mean ± standard deviation (SD) of triplicate determinations (technical triplicates) on three independent colonies of yeast transformants (biological replicates). For visual experiments the observed trends were fully consistent among the independent experiments and a representative example is shown. One sample *t* test was used for the statistical analysis of each strain compared with strain *myr*GFP or *myr*GFP-Cup1 under the specific conditions. Asterisks indicate the level of significance: *p < 0.05, **p < 0.01, and ***p < 0.001.

## Results and discussion

### Expression in yeast cells of *myr*GFP C-tagged with plant MTs

After entering the cell, free metal ions are buffered by a variety of anions present in the cytosol, such as phosphate and polyphosphate or by the metal-induced native MTs. If in excess, metal ions bind non-specifically to biomolecules, such as proteins, altering their function. We speculated that the presence of metal-binding ligands adjacent to the plasma membrane would ensure cation sequestration immediately after crossing the plasma membrane, thus preventing the potential deleterious effects of the free cations. To test this hypothesis, the cDNA of seven *Arabidopsis thaliana* MTs (*At*MT1a, *At*MT1c, *At*MT2a, *At*MT2b, *At*MT3, *At*MT4a and *At*MT4b) and four *Noccaea caerulescens* MTs (*Nc*MT1, *Nc*MT2a, *Nc*MT2b and *Nc*MT3) (S1 Table) were translationally fused to the C-terminus of a chimeric protein consisting of an N-terminal myristoylation sequence followed by the green fluorescent protein (*myr*GFP)(S2 Table). Proteins bearing this myristoylation sequence (MGCTVSTQTI) are targeted preponderantly to the plasma membrane [38]. In our case the expressed *myr*GFP-MT fusion proteins would be anchored by myristoylation to the inner leaflet of the lipid bilayer, while the GFP domain would act as a fluorescent linker, leaving the MT domain free to face the cytosol. As the primary goal of our study was to manipulate yeast cells towards increased accumulation of metal ions without developing the metal-related toxicity symptoms, we hypothesized that the metal ions would lose their innate toxicity if they encountered a metal binding protein (MT) immediately after penetrating the cell.

To test this possibility, we made use of a vector (pGREG596, [33]) that contains the DNA cassette for *myr*GFP under the control of the galactose-inducible *GAL1* yeast promoter [39] which allows the expression of a downstream gene *solely* when cells are grown on media containing galactose as carbon source (and not glucose).

**Fig 1:**
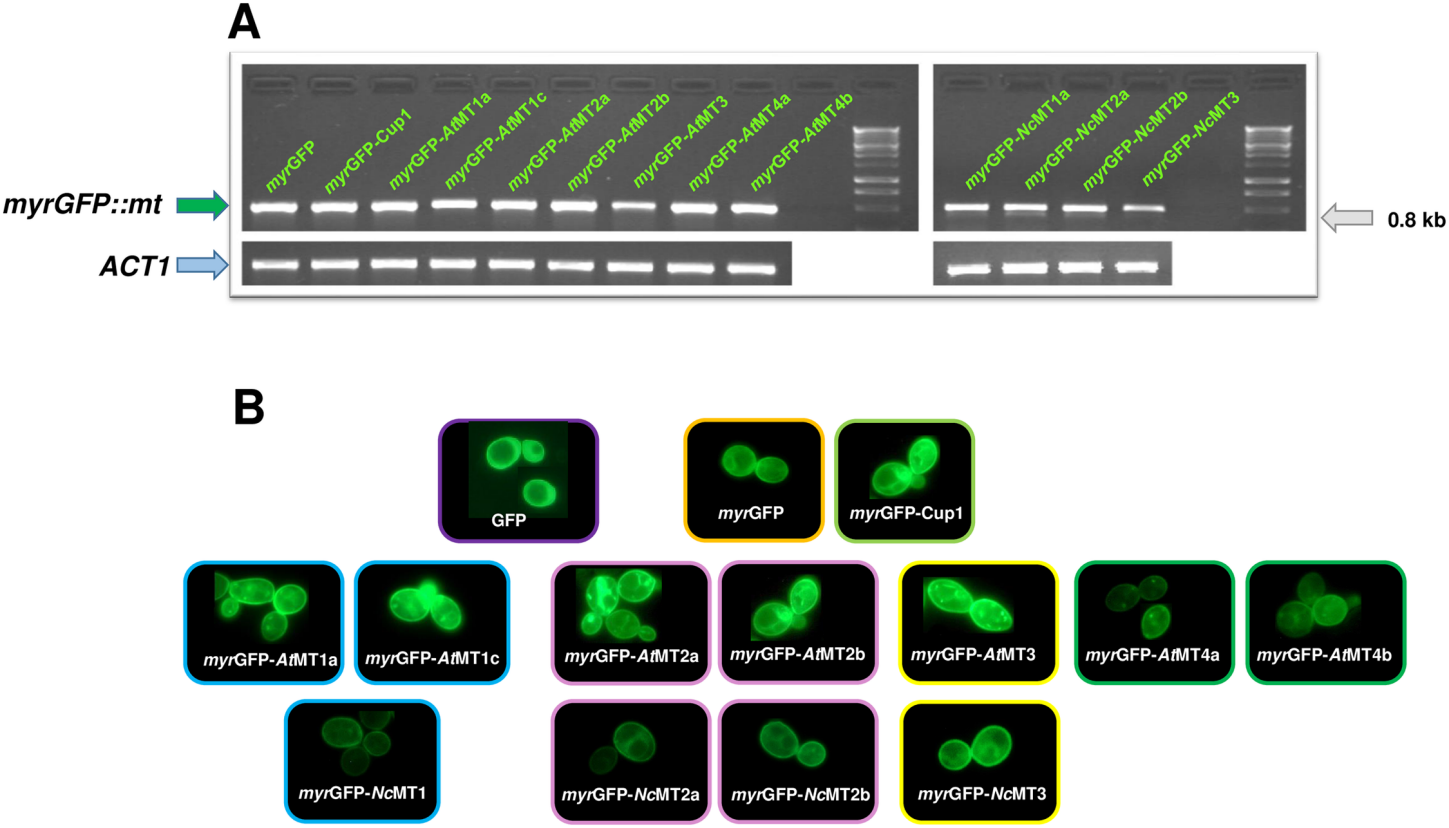
Expression of *myr*GFP-MTx in yeast cells. **A.** Expression of *myr*GFP-MTx was checked by agarose gel electrophoresis on amplicons obtained by Reverse-Transcription PCR made on RNA extracted from BY4741 cells transformed with pGRD-myrGFP::MTx series (Table 1) and grown in SGal-Ura as described in *Materials and methods*. *myrGFP*::*mt*, cDNA fragment containing *myrGFP* and 67 bp of the *MT* sequence. *ACT1*, fragment of actin gene (control). **B.** Cellular localization of *myr*GFP-MTx. Transgenic cells expressing myrGFP-MTx were prepared for visualization as described in *Materials and methods*. Live cell fluorescence revealed the concentration at the plasma membrane of the transgenic *myr*GFP-MTx studied. Cells expressing cytosolic GFP (up, left) were obtained by transforming yeast with pGREG600 [33], a plasmid harboring GFP under *GAL1* control. The experiments were done on at least three independent transformants, with similar results. For each strain, one representative example is shown.

The laboratory strain BY4741 was transformed with the pGRD-myrGFP::MTx plasmid series (Table 1); the transgenic strains were selected on SD-Ura medium and checked by colony PCR. Early log phase pre-cultures in liquid SD-Ura were washed and shifted to galactose-containing selective medium (SGal-Ura) for the induction of *myrGFP*::*MTx* expression. The transcription of the chimeric constructs was checked by reverse transcription PCR (RT-PCR) using primers that flanked the GFP C-terminus and the fragment situated approximately 60 bp downstream the *GFP*::*MTx* fusion (S3 Table). The RT-PCR amplicons analyzed by agarose gel electrophoresis revealed that all the chimeric *myrGFP*::*MTx* genes were transcribed (Fig 1A).

The production of fusion proteins was checked by fluorescence microscopy on cell samples taken every two hours from the galactose shift. It was noted that cells expressing *myr*GFP-MTx exhibited bright green fluorescence 4 hours after galactose shift (Fig 1B). The fluorescence concentrated at the cell periphery, suggesting that most of the *myr*GFP-MTx produced was targeted to the plasma membrane (Fig 1B). Fluorescence was detected when incubation in SGal-Ura continued overnight (checked up to 24 hours).

**Fig 2:**
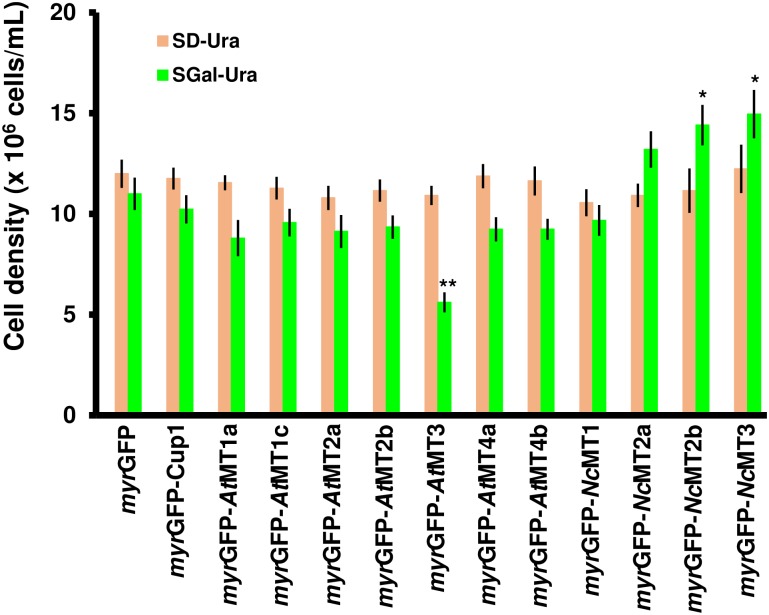
Growth of yeast cells expressing myrGFP-MTx. The BY4741 cells transformed with the pGRD-myrGFP::MTx series were shifted to SGal-Ura at density 1 x 10^6^ cells/mL (OD_600_ = 0.1) for transgene induction, as described in *Materials and methods*. The growth was determined for each strain spectrophotometrically (OD_600_) 24 h after the galactose shift. Values are means ± standard deviation of three independent data. Asterisks indicate that the mean of the *myr*GFP-MTx strain is significantly different from the mean of the *myr*GFP control under the same conditions, according to one sample *t* test. *p < 0.05, **p < 0.01.

To overrule the eventual toxicity of *myr*GFP-MTx expression, its effect on the growth of the transgenic lines was determined (Fig 2). Compared with the control *myr*GFP, the expression of *myr*GFP-MT_x_ was not deleterious to cell growth, with the exception of *myr*GFP-*At*MT3. In this case, albeit viable (S2A Fig), the cells grew slowly, having an unusually high doubling time. In contrast, cells expressing *myr*GFP-*Nc*MT2a, *myr*GFP-*Nc*MT2b, and especially *myr*GFP-*Nc*MT3 showed more robust growth compared to cells expressing control *myr*GFP (Fig 2).

### Accumulation of metals by yeast cells expressing *myr*GFP*-*MTx

#### Accumulation from media with low metal concentration

We first checked whether anchoring any of the plant MTs to the plasma membrane would trigger metal accumulation from media containing physiological concentrations of heavy metals. For this purpose MMe, a variant of the synthetic minimal medium for yeast [35] was used. MMe contained “traces” of the essential heavy metals (2 μM Mn(II) and Zn(II), 1 μM Cu(II), Co(II) and Ni(II), final concentration each). Since MTs are known to bind Cd(II), this non-essential metal was also used at 1 μM in MMe.

The metal accumulated by cells expressing various *myr*GFP-MTx was determined by ICP-MS on samples collected 10 hours after shifting the cells to MMe-Ura/Gal, and the results are presented in Table 2.

**Table 2:** Metal accumulation from minimal medium by yeast cells expressing *myr*GFP-MTx.

STRAIN	Cell metal content (nmoles/mg cell total protein)					
	Co	Cu	Mn	Ni	Zn	Cd
***myr*GFP**	0.4 ± 0.06	1.2 ± 0.4	1.4 ± 0.6	0.2 ± 0.04	4.2 ± 0.8	1.4 ± 0.3
***myr*GFP-Cup1**	5.9 ± 0.8	37.1 ± 2.9	12.8 ± 2.5	0.65 ± 0.1	35.8 ± 4.4	2.3 ± 0.4
***myr*GFP- *At*MT1a**	9.9 ± 1.4	48.8 ± 9.8	14.4 ± 2.3	0.34 ± 0.12	38.1 ± 5.2	3.8 ± 0.45
***myr*GFP- *At*MT1c**	6.68 ± 0.2	68.3 ± 12.8*	15.8 ± 2.8	0.38 ± 0.3	35.9 ± 3.4	1.5 ± 02
***myr*GFP- *At*MT2a**	1.74 ± 0.21	20.5 ± 1.3	11.5 ± 2.34	0.3 ± 0.12	28.3 ± 3.8	1.2 ± 0.2
***myr*GFP- *At*MT2b**	1.8 ± 0.12	16.72 ± 2.2	11.8 ± 2.4	0.56 ± 0.1	47.7 ± 5.4	1.5 ± 0.3
***myr*GFP- *At*MT3**	17.2 ± 2.2	60.1 ± 6.1*	18.2 ± 2.4	1.98 ± 0.8	403.7 ± 31.5**	16.4 ± 2.5*
***myr*GFP- *At*MT4a**	53.1 ± 12*	64.3 ± 5.8*	6.8 ± 0.9	0.9 ± 0.2	24.1 ± 2.3	1.8 ± 0.4
***myr*GFP- *At*MT4b**	48.7 ± 8.2*	52.4 ± 5.2	8.9 ± 1.4	0.98 ± 0.14	10.9 ± 1.4	1.1 ± 0.2
***myr*GFP- *Nc*MT1**	3.84 ± 0.9	58.1 ± 3.9*	23.3 ± 3.9	0.3 ± 0.1	37.2 ± 4.4	1.4 ± 0.2
***myr*GFP- *Nc*MT2a**	50.6 ± 4.4**	45.3 ± 4.2	38.8 ± 3.9**	3.2 ± 0.4**	438.2 ± 6.5***	1.6 ± 0.3
***myr*GFP- *Nc*MT2b**	60.9 ± 6.2**	38.5 ± 3.3	40.8 ± 4.2**	6.8 ± 1.2*	618 ± 89.8**	0.9 ± 0.2
***myr*GFP- *Nc*MT3**	15.8 ± 0.92	38.72 ± 3.7	19 ± 2.4	2.8 ± 0.7*	488.7 ± 37.5**	9.6 ± 1.2**

Early-log phase yeast cells transformed with the pGRD series (Table 1) were grown and shifted to MMe-Ura/Gal as described in *Materials and methods*. The minimal medium contained several essential metals (2 μM Mn(II) and Zn(II); 1 μM Co(II), Cu(II), Ni(II) and Cu(II)) and the non-essential metal Cd(II) (1μM). After 10 hours’ incubation (30°C, 200 rpm) the cells were harvested and processed for multielemental analysis (ICP-MS). Each determination was done in triplicate on approximately 10^8^ cells from three different transformants. Results are given as mean ± standard deviation. The transgenic strains accumulated metal ions significantly more than the control strain *myr*GFP, in most cases. Values significantly higher than the amount of the metal accumulated by *myr*GFP-Cup1, according to one sample *t* test, are shown in bold letters and marked with asterisks:

*p < 0.05,

**p < 0.01,

***p < 0.001.

The data revealed that all transgenic lines accumulated one or more metals from the MMe when compared to control *myr*GFP and this increased accumulation could be attributed to MT expression. Practically all transgenic lines accumulated Cu(II) significantly more than the control *myr*GFP strain (*p* < 0.05), with the highest accumulation being recorded for the strain expressing *myr*GFP-*At*MT1c. This did not come as a surprise, since all MT have a natural tendency to coordinate Cu(I) [40]. Functionally, the yeast MT, Cup1, is classified as the most restrictive Cu(I)-thionein [40], but the Cu(II) accumulation for *myr*GFP-*At*MT1c was almost doubled in comparison to *myr*GFP-Cup1. Interestingly, *myr*GFP-*At*MT1a differs from *myr*GFP-*At*MT1c by only two amino acids (S1B Fig), and although it accumulated less Cu(II) than the latter, it clearly accumulated more Cu(II) than *myr*GFP-Cup1. A similar behavior was noticed for *myr*GFP-*Nc*MT1, and also for *myr*GFP-*At*MT4a/b. Zn(II), another metal preferred by MTs, significantly accumulated in cells expressing *myr*GFP-*Nc*MT2a/2b and *myr*GFP-*Nc*MT3 (more than 100 times compared to cells expressing control *myr*GFP). In contrast, accumulation of Cd(II), chemically similar to Zn(II), occurred only in cells expressing *myr*GFP-MTx of subfamily 3 and it was rather modest, Cd(II) concentration in the medium being too low to be taken up efficiently by the innate yeast metal transporters. Remarkably Co(II), which is not a natural substrate of MTs, significantly accumulated in strains expressing *myr*GFP-*At*MT4a/4b and *myr*GFP-*Nc*MT2a/2b, the latter two also accumulating Mn(II). Notably, the sensitive strain expressing *myr*GFP-*At*MT3 accumulated significant amounts of Cu(II), Zn(II) and Cd(II) but also Co(II) and Mn(II), a possible explanation for the poor growth of this strain. Among the metals present in the medium, Ni(II) was least accumulated by all transgenic strains, and this was in contrast to the chemically-similar Co(II). Further studies are needed to establish whether the low Ni(II) accumulation was determined by its low affinity to MTs, or simply by the low Ni(II) translocation across the plasma membrane. Nevertheless, the accumulation of Ni(II) by *myr*GFP-*Nc*MT2a/2b and *myr*GFP-*Nc*MT3, even though modest, was more than 10 times higher when compared to the control *myr*GFP.

#### Accumulation from media supplemented with surplus heavy metals

For bioremediation purposes, it is important to obtain strains which accumulate metals under high concentration conditions, without developing toxicity symptoms. Therefore, we further determined the accumulative potential of our transgenic *myr*GFP-MTx strains under higher-than-normal metal concentrations. It was noticed that metal accumulation in galactose-containing media reached a plateau following 1–2 h of metal exposure; this is why metal accumulation by cells expressing *myr*GFP-MTx was determined 2 hours after the shift to metal-surplus media. The highest metal concentrations which were not deleterious to the control strain *myr*GFP were selected for the accumulation assays under metal surplus. These concentrations were set to 0.5 mM for Co(II), Cu(II) and Ni(II), 1 mM for Zn(II) and Mn(II), and 0.05 mM for Cd(II) and corresponded to the maximum concentrations which allowed a cell viability that was higher than 75% (S2A Fig), following exposure to metal for 2 hours (the time necessary to reach the metal accumulation plateau) or 20 hours (the time necessary to reach stationary phase). At these concentrations, the half-life (the time after which more than 50% of the cells lose viability) of all transgenic strains was longer than 24 hours (data not shown). At higher metal concentrations, cells expressing *myr*GFP-MTx had higher viabilities than control *myr*GFP (S2B Fig), suggesting that MTs expression had a protective effect against metal surplus.

The growth of the transgenic cells in the presence of surplus metals was determined after 20 h of exposure, a time which allowed cells to adapt to stress conditions and to proliferate. Metal accumulation determined after 20 h exposure did not differ significantly from the values obtained for accumulation after 2 h exposure (data not shown). The metals investigated are presented further in alphabetical order, and not based on their innate affinity for MTs.

**Fig 3:**
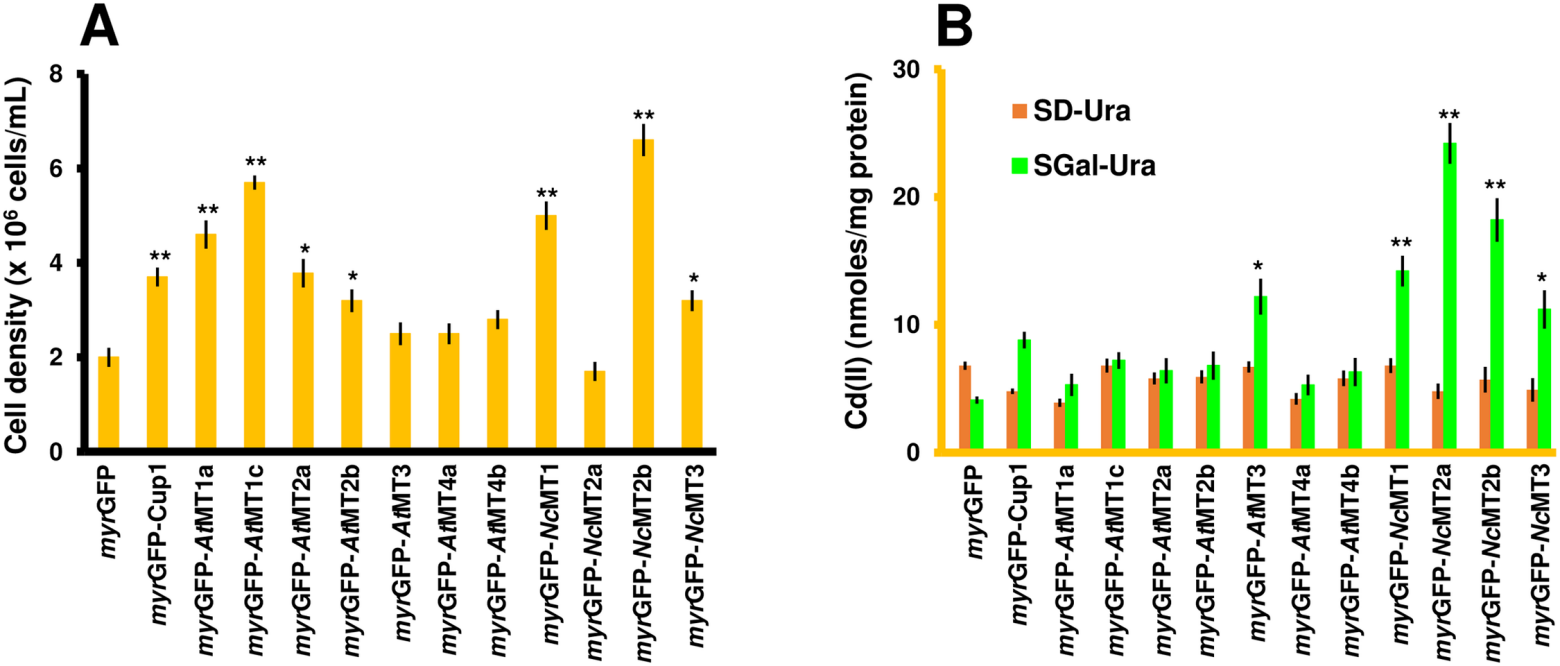
Cd(II) accumulation by yeast cells expressing *myr*GFP-MTx. Early log phase growing cells transformed with pGRD-myrGFP::MTx series were shifted to SGal-Ura for transgene induction as described in *Materials and methods*. Four hours after the galactose shift, CdCl_2_ was added (0.05 mM final concentration) **A. Growth of yeast cells expressing *myr*GFP-MTx under Cd(II) surplus.** The cell growth was determined for each strain spectrophotometrically (OD_600_) 20 h after adding the metal salt. **B. Cd(II) accumulation.** Cells were exposed to Cd(II) for 2 hours (30°C, 200 rpm) before being processed for metal assay by ICP-MS. The accumulated metal was normalized to cell total protein. Values are mean ± standard deviation of three independent data. Asterisks indicate that the mean of the *myr*GFP-MTx strain was significantly different from the mean of the *myr*GFP control under the same conditions, according to one sample *t* test. *p < 0.05, **p < 0.01.

Cd(II) It was noted that all strains expressing *myr*GFP-MTx grew considerably better in the presence of excess Cd(II) than the control *myr*GFP, except *myr*GFP-*Nc*MT2a (Fig 3A). This observation suggested that the very toxic Cd(II) ions are sequestered by the transgenic MTs in a less toxic form. Cd(II) accumulated significantly in strains expressing *myr*GFP-*At*MT3, *myr*GFP-*Nc*MT1, *myr*GFP-*Nc*MT2a/b and *myr*GFP-*Nc*MT3 (Fig 3B), but the expected combination for a potential bioremediator (robust growth and high accumulation) was only obtained for *myr*GFP-*Nc*MT1, *myr*GFP-*Nc*MT2b and *myr*GFP-*Nc*MT3.

Even though the maximum accumulation was recorded for *myr*GFP-*Nc*MT2a (Fig 3B), the growth of this strain was affected by the surplus Cd(II) (Fig 3A). The best tolerance/accumulation ratio was noted for the *myr*GFP-*Nc*MT2b strain, making it a good candidate for further studies on Cd(II) bioremediation.

**Fig 4:**
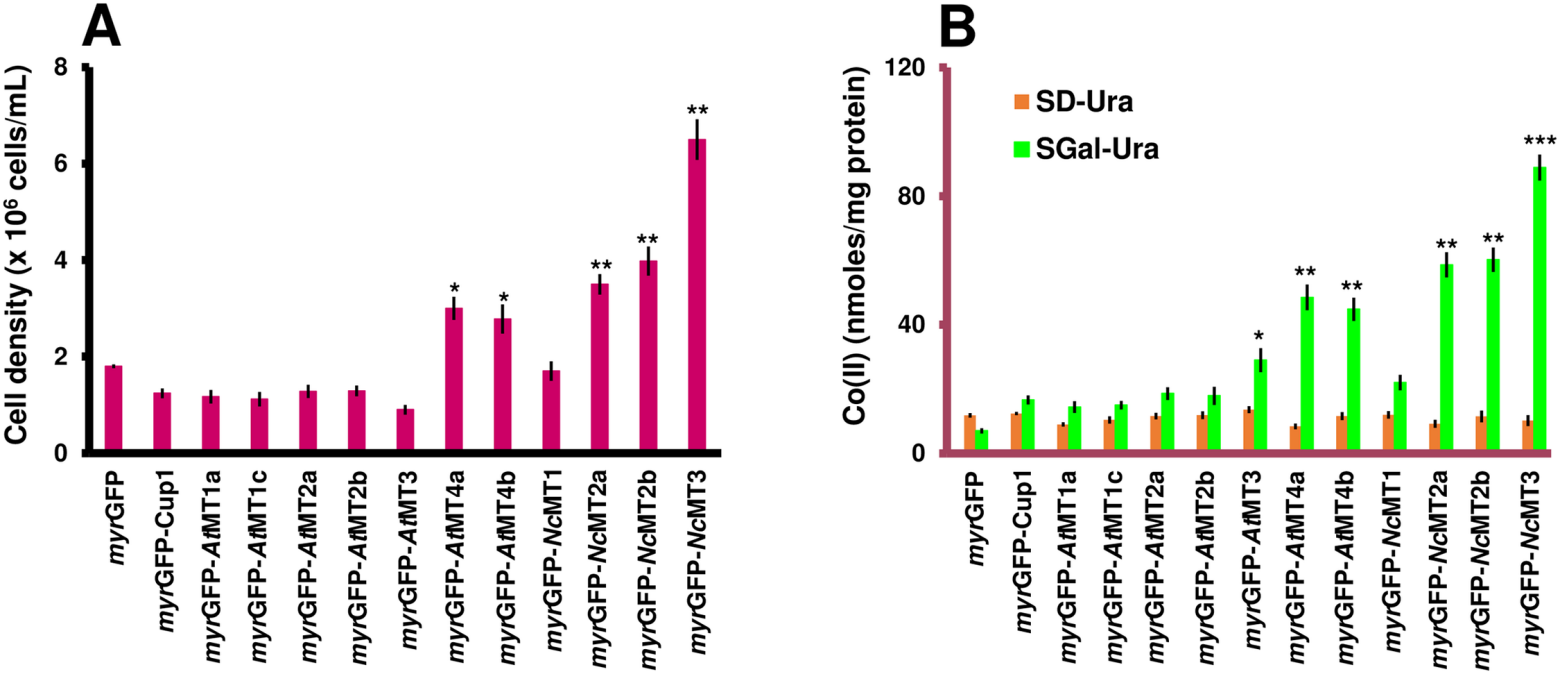
Co(II) accumulation by yeast cells expressing *myr*GFP-MTx. Yeast cells were manipulated as described in Fig 3, except that CoCl_2_ was added at final concentration 0.5 mM. **A. Growth of yeast cells expressing *myr*GFP-MTx under Co(II) surplus.** The cell growth was determined for each strain spectrophotometrically (OD_600_) 20 h after adding the metal salt. **B. Co(II) accumulation.** Cells were exposed to Co(II) for 2 hours (30°C, 200 rpm) before being processed for metal assay by ICP-MS. Accumulated metal was normalized to cell total protein. Values are mean ± standard deviation of three independent data. Asterisks indicate that the mean of the *myr*GFP-MTx strain was significantly different from the mean of the myrGFP control under the same conditions, according to one sample *t* test. *p < 0.05, **p < 0.01, ***p < 0.001.

Co(II) Co(II) impaired the growth of cells expressing *myr*GFP-Cup1, *myr*GFP-*At*MTs of subfamilies 1, 2 and 3 and *myr*GFP-*Nc*MT1 more than that of the *myr*GFP control, but not of *myr*GFP-*At*MT4a/b, *myr*GFP-*Nc*MT2a/b or *myr*GFP-*Nc*MT3 (Fig 4A). As in the case of the accumulation from MMe, containing “trace” metal concentrations, the pairs *myr*GFP-*At*MT4a/4b and *myr*GFP-*Nc*MT2a/2b also accumulated Co(II) under surplus condition (Fig 4B). However, the accumulation of Co(II) under the surplus condition was not significantly higher than that under the low-concentration condition, suggesting that a saturation limit was reached in both cases.

Maximum Co(II) accumulation was recorded for *myr*GFP-*Nc*MT3 (Fig 4B), which also showed robust growth under high Co(II) conditions (Fig 4A), making this strain a good candidate for Co(II) bioremediation.

**Fig 5:**
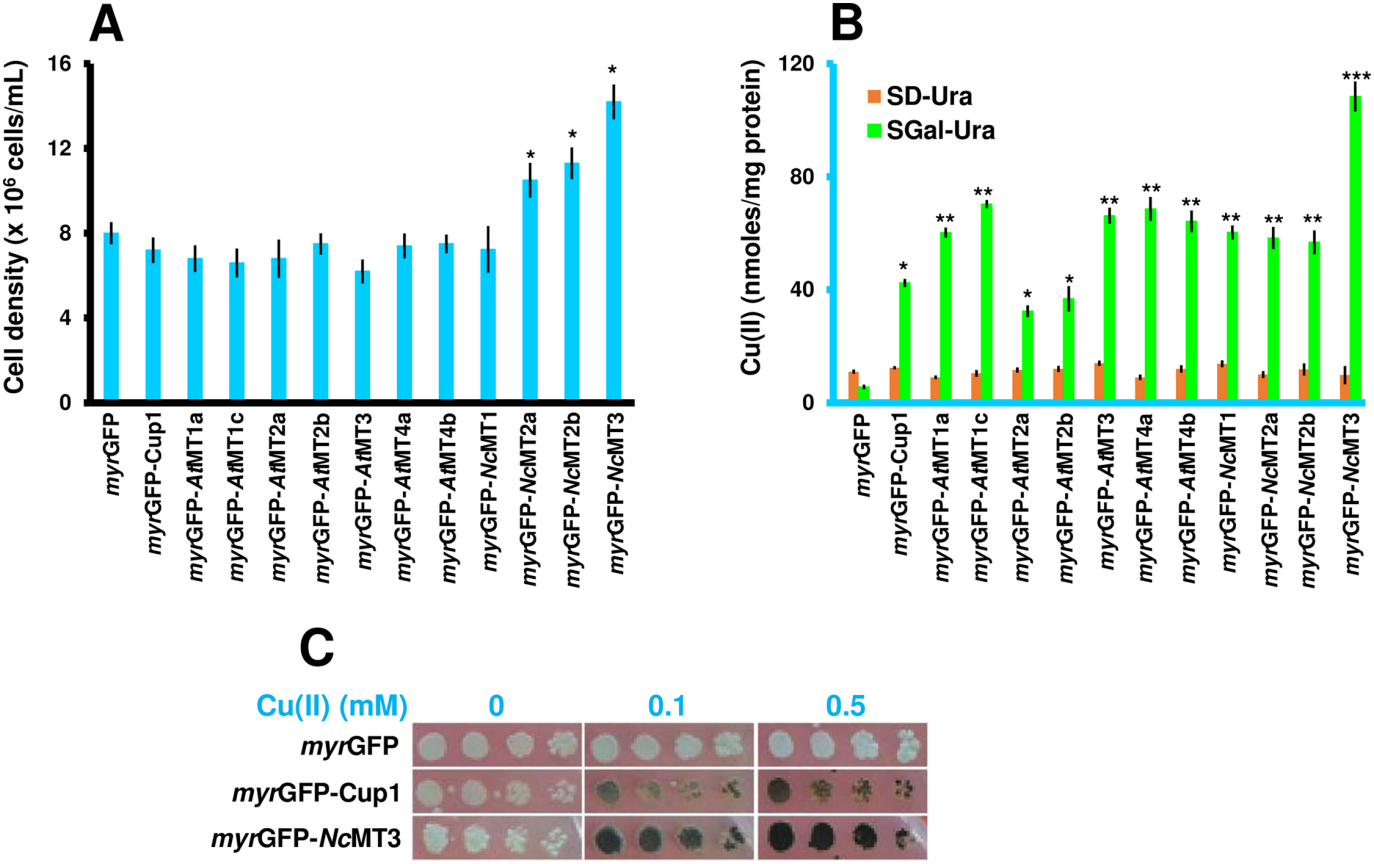
Cu(II) accumulation by yeast cells expressing *myr*GFP-MTx. Yeast cells were manipulated as described in Fig 3, except that CuCl_2_ was added at a final concentration of 0.5 mM. **A. Growth of yeast cells expressing *myr*GFP-MTx under Cu(II) surplus.** The cell growth was determined for each strain spectrophotometrically (OD_600_) 20 h after adding the metal salt. **B. Cu(II) accumulation.** Cells were exposed to Cu(II) for 2 hours (30°C, 200 rpm) before being processed for metal assay by ICP-MS. Accumulated metal was normalized to cell total protein. Values are mean ± standard deviation of three independent data. Asterisks indicate that the mean of the *myr*GFP-MTx strain was significantly different from the mean of the *myr*GFP control under the same conditions, according to one sample t test. *p < 0.05, **p < 0.01, ***p < 0.001.**C. Cu(II) accumulation from solid medium.** Cells were 10-fold serially diluted in a multi-well plate and stamped with a replicator on SGal-Ura plates containing the specified concentrations of Cu(II). Plates were photographed after 6 days’ incubation at 30°C. One representative plate is shown out of three similar experiments.

Cu(II) The growth of the yeast cells expressing *myr*GFP-MTx was not significantly altered by Cu(II) addition when compared to the control *myr*GFP, with the exception of *myr*GFP-*Nc*MT2a/b and *myr*GFP-*Nc*MT3 whose expression supported better growth under Cu(II) surplus (Fig 5A). All transgenic strains accumulated more Cu(II) than the control strain expressing *myr*GFP (Fig 5B). Nevertheless, the only strain which accumulated *significantly* more Cu(II) under high concentration conditions compared to accumulation under trace Cu(II) conditions was strain *myr*GFP-*Nc*MT3 (Fig 5B), which also showed robust growth under the same conditions (Fig 5A).

Cu(II) accumulation could be also visualized with naked eye in transgenic cells grown on solid media, as the cells changed color following prolonged incubation (Fig 5C).

**Fig 6:**
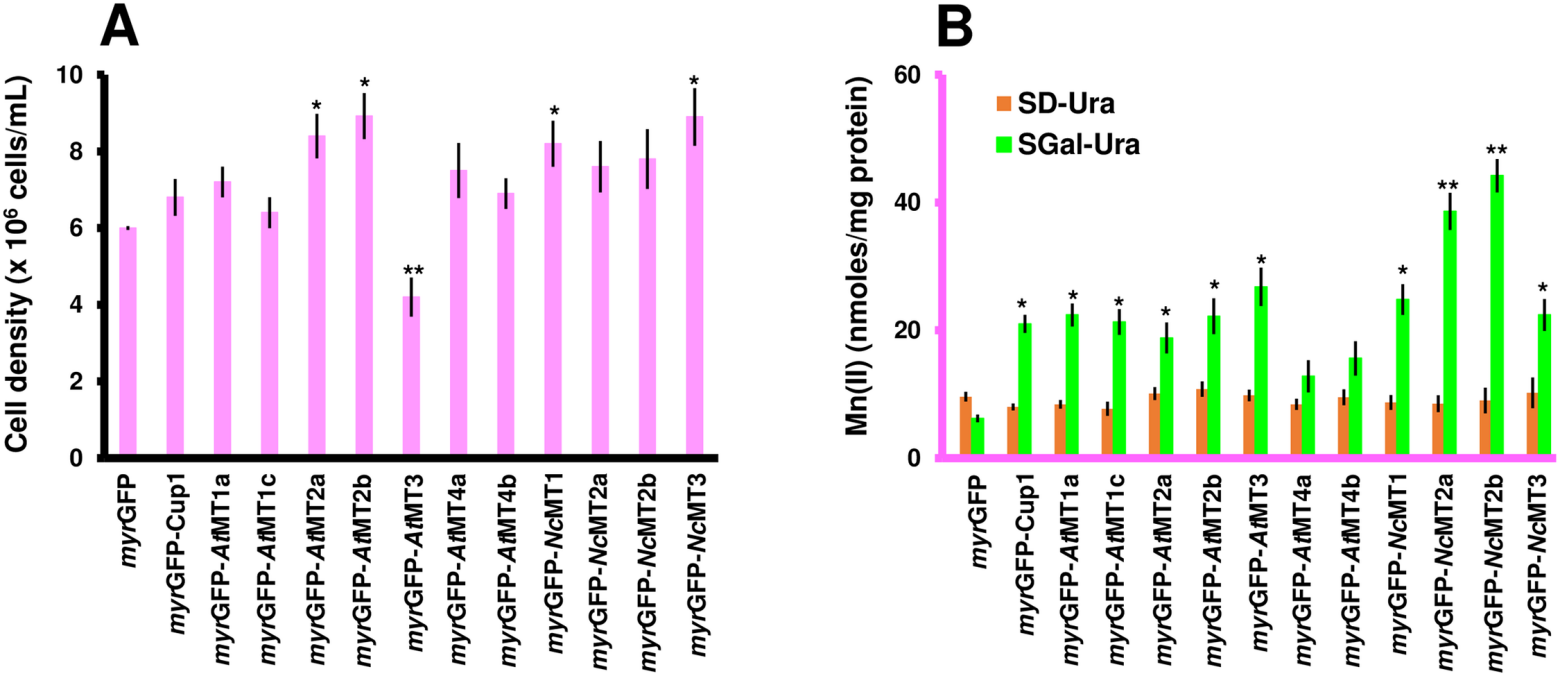
Mn(II) accumulation by yeast cells expressing myrGFP-MTx. Yeast cells were manipulated as described in Fig 3, except that MnCl_2_ was added at a final concentration of 1 mM. **A. Growth of yeast cells expressing *myr*GFP-MTx under Mn(II) surplus.** The cell growth was determined for each strain spectrophotometrically (OD_600_) 20 h after adding the metal. **B. Mn(II) accumulation.** Cells were exposed to Mn(II) for 2 hours (30°C, 200 rpm) before being processed for metal assay by ICP-MS. Accumulated metal was normalized to cell total protein. Values are mean ± standard deviation of three independent data. Asterisks indicate that the mean of the *myr*GFP-MT strain is significantly different from the mean of the *myr*GFP control under the same conditions, according to one sample *t* test. *p < 0.05, **p < 0.01.

Mn(II) As seen in Fig 6A, Mn(II) hardly affected the growth of most transgenic cells expressing *myr*GFP-MTx in comparison to *myr*GFP (Fig 6A). Mn(II) is not a natural substrate of MTs, but it was significantly taken up from normal medium by strains expressing *myr*GFP-*Nc*MT2a/2b (Table 2). Under surplus conditions these two strains also accumulated Mn(II), but the accumulation was not significantly higher compared to the low concentration conditions, probably due to saturation (Fig 6B).

Although Mn(II) accumulation was noticed in most of the other transgenic strains tested (Fig 6B), the accumulation under high Mn(II) did not differ significantly when compared to the low concentration conditions.

**Fig 7:**
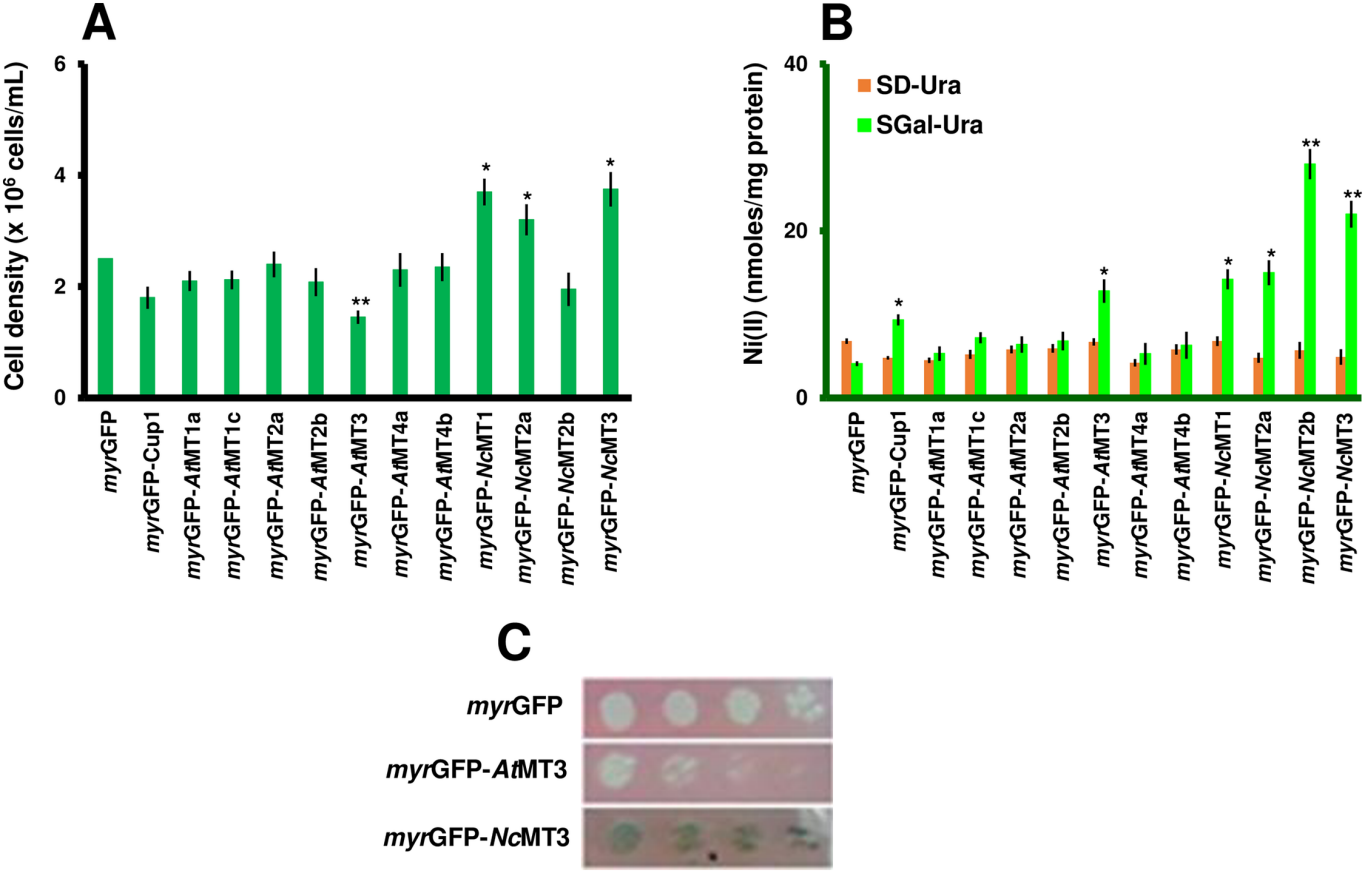
Ni(II) accumulation by yeast cells expressing *myr*GFP-MTx. Yeast cells were manipulated as described in Fig 3, except that NiCl_2_ was added at final concentration 0.5 mM. **A. Growth of yeast cells expressing *myr*GFP-MTx under Ni(II) surplus.** The cell growth was determined for each strain spectrophotometrically (OD_600_) 20 h after adding the metal. **B. Ni(II) accumulation.** Cells were exposed to Ni(II) for 2 hours (30°C, 200 rpm) before being processed for metal assay by ICP-MS. Accumulated metal was normalized to cell total protein. Values are mean ± standard deviation of three independent data. Asterisks indicate that the mean of the *myr*GFP-MT line is significantly different from the mean of the myrGFP control under the same conditions, according to one sample *t* test. *p < 0.05, **p < 0.01. **C. Ni(II) accumulation from solid medium.** Cells were 10-fold serially diluted in a multi-well plate and stamped with a replicator on SGal-Ura plates containing 2 mM NiCl_2_. Plates were photographed after 6 days’ incubation at 30°C. One representative plate is shown out of three similar experiments.

Ni(II) Under a high Ni(II) concentration only cells expressing *myr*GFP-*Nc*MT1, *myr*GFP-*Nc*MT2a and *myr*GFP-*Nc*MT3 grew better than the *myr*GFP control strain (Fig 7A). As Ni(II) is known as one of the most recalcitrant metals in terms of removal from contaminated sites [1], obtaining an efficient Ni(II)-hyperaccumulating organism would represent a major asset for bioremediation techniques. Ni(II) accumulation by *myr*GFP-MTx under normal conditions was rather modest, the highest value being recorded for the strain expressing *myr*GFP-*Nc*MT2b (Table 2). This strain also accumulated most Ni(II) under surplus conditions (Fig 7B), but its growth was not robust (Fig 7A). In fact, it was interesting to notice the apparent opposite behavior towards Ni(II) of cells expressing *myr*GFP-*Nc*MT2a and *myr*GFP-*Nc*MT2b (Fig 7A and 7B). Whether the small changes in the amino acid sequence (S1C Fig) are responsible for the different accumulation capacity of the two transgenic MTs are issues to be investigated in the future.

Although the strain expressing *myr*GFP-*Nc*MT3 accumulated less Ni(II) than the strain expressing *myr*GFP-*Nc*MT2b (Fig 7B), the former is a more suitable candidate for further studies due to its robust growth under high Ni(II) (Fig 7A). Moreover, it was noted that cells expressing *myr*GFP-*Nc*MT3 gradually turned green after prolonged incubation on solid medium supplemented with Ni(II), probably due to continuous accumulation of this cation (Fig 7C).

Zn(II) It was intriguing to notice that under surplus conditions, Zn(II) accumulation by *myr*GFP-MTx strains did not differ significantly from Zn(II) accumulation under normal concentration conditions (data not shown). Zn(II) is an essential element supplied from the environment by two plasma membrane transporters: Zrt1 (high affinity) and Zrt2 (low affinity), both upregulated by low cellular Zn(II) [41]. It is tempting to speculate that in cells expressing *myr*GFP-MTx under “normal” conditions (i. e., MMe), the Zn(II) ions entering the cell were readily sequestered by the transgenic MTs, resulting in intracellular Zn(II) depletion and triggering Zrt1/2 activation. The result of this series of events would be an unusually high accumulation of Zn(II) by strains such as *myr*GFP-*At*MT3, *myr*GFP-*Nc*MT2a/b and *myr*GFP-*Nc*MT3 (Table 2). The Zn(II) accumulation would continue until *myr*GFP*-*MTx reached saturation, explaining why increasing the Zn(II) concentration in the growth environment would have only a modest effect upon Zn(II) accumulation.

## Conclusions

*Saccharomyces cerevisiae* has often been used to express plant methallothioneins (MTs) either for functional elucidation through complementation studies [24, 28–31] or for their metal-binding abilities [32, 42–45]. While most of the studies concerning heterologous expression of MTs in yeast focus on canonical MT substrates, our work encompassed a greater variety of both natural and non-canonical MT substrates, all in parallel settings. Aiming at obtaining heavy metal accumulating cells we expressed seven MTs from the non-accumulator plant *A*. *thaliana* and four MTs from the hyperaccumulator plant *N*. *caerulescens* in *S*. *cerevisiae*. Unlike any other study so far, the MTs were targeted to the inner face of the plasma membrane via a myristoylation sequence that was N-fused to GFP. This approach has the advantage that the osmotically-free metal ions that reach the cytosol are readily sequestered by the anchored MTs, increasing the chances to obtain a hyperaccumulating strain with increased tolerance to otherwise toxic heavy metals.

With the exception of *myr*GFP-*At*MT3, the expression of none of the other *myr*GFP-MTx affected yeast growth negatively, allowing high accumulation of metals from environments containing either low or high metal concentrations. It is of note that, in most cases, the transgenic strains which expressed these plant MTs had higher accumulative power than the strain expressing *myr*GFP-Cup1 (yeast MT).

All transgenic strains expressing *myr*GFP-MTx accumulated Cu(II) under low concentration conditions and the possibility to use these strains to extract similar ions such as Ag(I) is under investigation. Of note are also the constructs which allowed the accumulation from “normal” media of metals less usual in MT-related studies: Co(II) (*myr*GFP-*At*MT4a/b, *myr*GFP-*Nc*MT2a/b) or Mn(II) (*myr*GFP-*Nc*MT2a/b). The reasons for such preferences are now being considered by molecular modelling, starting from the structural differences between MT classes, but also from the differences between MTs within the same subfamily.

It was interesting to notice that compared to *myr*GFP and *myr*GFP-Cup1 we obtained one poorly growing (*myr*GFP-*At*MT3) and one robust growing strain (*myr*GFP-*Nc*MT3), both belonging to the same family of plant MTs. Remarkably, *myr*GFP-*At*MT3 accumulated almost indiscriminately all the metal ions tested under low concentration conditions, a possible explanation for its reduced growth rates. The viability of this strain was however unusually high, as cells survived for days under high metal surplus without proliferating, but exhibiting continuous fluorescence at the plasma membrane level (data not shown). Due to these characteristics the *myr*GFP-*At*MT3 strain is an interesting candidate for further studies. In contrast, *myr*GFP-*Nc*MT3 seemed the most suitable strain for bioremediation studies, as it exhibited robust growth, metal tolerance and high accumulative capacity under high concentration conditions for the metals tested in this study.

## Supporting information

S1 Fig Multiple amino acid sequence alignments of metallothioneins cloned for expression in yeast (sequence names with _X; the first methionine has been removed) and their reference protein sequences. **A.** *Sc*CUP1 (P0CX80), metallothionein from *Saccharomyces cerevisiae*. **B.** Subfamily 1 of *Arabidopsis thaliana* Col-0 metallothioneins *At*MT1a (P43392) and *At*MT1c (Q38804) and the *Noccaea caerulescens Nc*MT1 metallothionein from the ecotypes Ganges (NcMT1_G, AAX40656) and Prayon (NcMT1_P, AY486003). Only one *Nc*MT1 ecotype was used to clone *Nc*MT1. **C.** Subfamily 2 metallothioneins *At*MT2a (P25860) and *At*MT2b (Q38805) from *A*. *thaliana* Col-0 and *Nc*MT2a (ACR46970) and *Nc*MT2b (ACR46961) from *N*. *caerulescens* La Calamine. **D.** Subfamily 3 metallothionein *At*MT3 (O22433) from *A*. *thaliana* Col-0 and NcMT3 (ACR46965) from *N*. *caerulescens* La Calamine. **E.** The two *A*. *thaliana* Col-0 metallothioneins of subfamily 4, which might be present as to polypeptides each (generated by alternative splicing): AtMT4a (p1: P93746, p2: F4ILY7) and AtMT4b (p1: Q42377, p2: F4ILI2). Cysteines are highlighted in blue.
(TIF)

S2 Fig Effect of metal exposure on the viability of *myr*GFP-MT-expressing cells. Early log phase growing cells transformed with pGRD-myrGFP::MTx series were shifted to SGal-Ura for transgene induction. Four hours after the galactose shift, MeCl_2_ was added at the indicated concentration and samples were harvested for viability assay by methylene blue staining. Cell viability was expressed as percentage of live cells within a whole population. Viability was examined for at least 300 cells from one biological replicate. Viable cells were colorless, and dead cells were blue. Values are means ± standard deviation of three independent data. **A.** Maximum metal concentrations which did not significantly alter cell viability. **B.** At higher metal concentrations, viability of cells expressing *myr*GFP-MTx is less affected compared to control *myr*GFP.
(TIF)

S1 Table Primers used to clone plant metallothionein cDNAs. The primers were used to amplify plant MTs cDNAs from *Arabidopsis thaliana (At)* or *Noccaea caerulescens (Nc)* cDNAs. The MT gene *CUP1* from *Saccharomyces cerevisiae (Sc)* is intronless and was amplified from genomic DNA. The primers used introduced restriction sites suitable for subsequent subcloning of the amplified fragments into yeast vectors. To amplify the *myr*GFP control, pGREG596 was used as template. The map of plasmid pGREG596 can be found at (http://web.uni-frankfurt.de/fb15/mikro/euroscarf/data/pGREG.html).
(DOCX)

S2 Table Amino acid sequence of the chimeric metallothioneins (*myr*GFP-MTx) expressed in yeast cells. (DOCX)

S3 Table Primers used to verify the transcription of the transgenic myrGFP::MT in yeast cells by reverse transcription-PCR (RT-PCR). The forward primer (universal) overlapped nucleotides 8–27 of myrGFP fragment, while the reverse primer complemented the fragment 48–67 of each fused MT cDNA.
(DOCX)

## References

[1] Bradl H, editor. Heavy Metals in the Environment: Origin, Interaction and Remediation Volume 6 London: Academic Press, 2002.

[2] He ZL, Yang XE, Stoffella PJ. 2005 Trace elements in agroecosystems and impacts on the environment. J Trace Elem Med Biol. 2005; 19: 125–140. 10.1016/j.jtemb.2005.02.010 16325528

[3] Verma N, Sharma R. Bioremediation of Toxic Heavy Metals: A Patent Review. Recent Pat Biotechnol. 2017 1 11. Forthcoming 10.2174/1872208311666170111111631 28078980

[4] Mishra GK. Microbes in Heavy Metal Remediation: A Review on Current Trends and Patents. Recent Pat Biotechnol. 2017 1 20. Forthcoming 10.2174/1872208311666170120121025 28116999

[5] Bolan N, Kunhikrishnan A, Thangarajan R, Kumpiene J, Park J, Makino T, et al Remediation of heavy metal(loid)s contaminated soils—to mobilize or to immobilize? J Hazard Mater. 2014; 266: 141–166. 10.1016/j.jhazmat.2013.12.018 24394669

[6] Gaur N, Flora G, Yadav M, Tiwari A. A review with recent advancements on bioremediation-based abolition of heavy metals. Environ Sci Process Impacts. 2014; 16: 180–193. 10.1039/c3em00491k 24362580

[7] Soares EV, Soares HM. Cleanup of industrial effluents containing heavy metals: a new opportunity of valorising the biomass produced by brewing industry. Appl Microbiol Biotechnol. 2013; 97: 6667–6675 10.1007/s00253-013-5063-y 23824444

[8] Machado MD, Santos MS, Gouveia C, Soares HM, Soares EV. Removal of heavy metals using a brewer’s yeast strain of *Saccharomyces cerevisiae*: the flocculation as a separation process. Bioresours Technol. 2008; 99: 2107–2115. 10.1016/j.biortech.2007.05.047 17631999

[9] Machado MD, Janssens S, Soares HM, Soares EV. Removal of heavy metals using a brewer’s yeast strain of *Saccharomyces cerevisiae*: advantages of using dead biomass. J Appl Biotech. 2009; 106: 1792–1804. 10.1111/j.1365-2672.2009.04170.x 19245404

[10] Kambe-Honjoh H, Ohsumi K, Shimoi H, Nakajima H, Kitamoto K. Molecular breeding of yeast with higher metal-adsorption capacity by expression of histidine-repeat insertion in the protein anchored to the cell wall. J Gen Appl Microbiol. 2000; 46: 113–117. 12483584 10.2323/jgam.46.113

[11] Kuroda K, Shibasaki S, Ueda M, Tanaka A. Cell surface engineered yeast displaying a histidine oligopeptide (hexa-His) has enhanced adsorption of and tolerance to heavy metal ions. Appl Microbiol Biotechnol. 2001; 57: 697–701. 11778880 10.1007/s002530100813

[12] Kuroda K, Ueda M, Shibasaki S, Tanaka A. Cell surface engineered yeast with ability to bind, and self-aggregate in response to, copper ion. Appl Microbiol Biotechnol. 2002; 59: 259–264. 10.1007/s00253-002-1014-8 12111155

[13] Kuroda K, Ueda M. Bioadsorption of cadmium ion by cell surface-engineered yeasts displaying metallothionein and hexa-His. Appl Microbiol Biotechnol. 2003; 63: 182–186. 10.1007/s00253-003-1399-z 12898063

[14] Kuroda K, Ueda M. Effective display of metallothionein tandem repeats on the bioadsorption of cadmium ion. Appl Microbiol Biotechnol. 2006; 70: 458–463. 10.1007/s00253-005-0093-8 16091929

[15] Kotrba P, Rumi T. Surface display of metal fixation motifs of bacterial P1-type ATPase specifically promotes biosorption of Pb(2+) by *Saccharomyces cerevisiae*. Appl Environ Microbiol. 2010; 76:2 615–2622. 10.1128/AEM.01463-09 PMC2849214 20173062

[16] Kuroda K, Ueda M. Engineering of microorganisms towards recovery of rare metal ions. Appl Microbiol Biotechnol. 2010; 87: 53–60. 10.1007/s00253-010-2581-8 20393699

[17] Liu Z, Ho SH, Hasunuma T, Chang JS, Ren NQ, Kondo A. Recent advances in yeast cell-surface display technologies for waste biorefineries. Bioresour Technol. 2016; 215: 324–333. 10.1016/j.biortech.2016.03.132 27039354

[18] Ruta L, Paraschivescu C, Matache M, Avramescu S, Farcasanu IC. Removing heavy metals from synthetic effluents using "kamikaze" *Saccharomyces cerevisiae* cells. Appl Microbiol Biotechnol. 2010; 85: 763–771. 10.1007/s00253-009-2266-3 19795117

[19] Ofiteru AM, Ruta LL, Rotaru C, Dumitru I, Ene CD, Neagoe A, et al Overexpression of the *PHO84* gene causes heavy metal accumulation and induces Ire1p-dependent unfolded protein response in *Saccharomyces cerevisiae* cells. Appl Microbiol Biotechnol. 2012; 94: 425–435. 10.1007/s00253-011-3784-3 22207212

[20] Dürr G, Strayle J, Plemper R, Elbs S, Klee SK, Catty P, et al The medial-Golgi ion pump Pmr1 supplies the yeast secretory pathway with Ca^2+^ and Mn^2+^ required for glycosylation, sorting, and endoplasmic reticulum-associated protein degradation. Mol Biol Cell. 1998; 9: 1149–1162. 9571246 10.1091/mbc.9.5.1149 PMC25337

[21] Lauer Júnior CM, Bonatto D, Mielniczki-Pereira AA, Schuch AZ, Dias JF, Yoneama ML, et al The Pmr1 protein, the major yeast Ca2+-ATPase in the Golgi, regulates intracellular levels of the cadmium ion. FEMS Microbiol Lett. 2008; 285: 79–88. 10.1111/j.1574-6968.2008.01214.x 18510555

[22] Capdevila M, Atrian s. Metallothionein protein evolution: a miniassay. J Biol Inorg Chem. 2011; 16: 977–989. 10.1007/s00775-011-0798-3 21633816

[23] Vasak M. Metallothioneins: chemical and biological challenges. J Biol Inorg Chem. 2011; 975–976. 10.1007/s00775-011-0832-5 21904890

[24] Freisinger E. Structural features specific to plant metallothioneins. J Biol Inorg Chem. 2011; 16: 1035–1045. 10.1007/s00775-011-0801-z 21688177

[25] Hassinen VH, Tervahauta AI, Schat H, Kärenlampi SO. Plant metallothioneins—metal chelators with ROS scavenging activity? Plant Biol (Stuttg). 2011; 13: 225–232. 21309968 10.1111/j.1438-8677.2010.00398.x

[26] Butt TR, Sternberg EJ, Gorman JA, Clark P, Hamer D, Rosenberg M, et al Copper metallothionein of yeast, structure of the gene, and regulation of expression. Proc Natl Acad Sci U S A. 1984; 81: 3332–3336. 6374656 10.1073/pnas.81.11.3332 PMC345501

[27] Nevitt T, Ohrvik H, Thiele DJ. Charting the travels of copper in eukaryotes from yeast to mammals. Biochim Biophys Acta. 2012; 1823: 1580–1593. 10.1016/j.bbamcr.2012.02.011 22387373 PMC3392525

[28] Zhou J, Goldsbrough PB. Functional homologs of fungal metallothionein genes from *Arabidopsis*. Plant Cell. 1994; 6: 875–884. 10.1105/tpc.6.6.875 8061521 PMC160485

[29] Guo WJ, Meetam M, Goldsbrough PB. Examining the specific contributions of individual *Arabidopsis* metallothioneins to copper distribution and metal tolerance. Plant Physiol. 2008; 146: 1697–706. 10.1104/pp.108.115782 18287486 PMC2287344

[30] Roosens NH, Bernard C, Leplae R, Verbruggen N. Evidence for copper homeostasis function of metallothionein (MT3) in the hyperaccumulator *Thlaspi caerulescens*. FEBS Lett. 2004; 577: 9–16. 10.1016/j.febslet.2004.08.084 15527754

[31] Roosens NH, Leplae R, Bernard C, Verbruggen N. Variations in plant metallothioneins: the heavy metal hyperaccumulator *Thlaspi caerulescens* as a study case. Planta. 2005; 222: 716–729. 10.1007/s00425-005-0006-1 16052319

[32] Wei Q, Zhang H, Guo D, Ma S. Cell surface display of four types of *Solanum nigrum* metallothionein on *Saccharomyces cerevisiae* for biosorption of cadmium. J Microbiol Biotechnol. 2016; 26: 846–853. 10.4014/jmb.1512.12041 26838339

[33] Jansen G, Wu C, Schade B, Thomas DY, Whiteway M. Drag&Drop cloning in yeast. Gene. 2005; 344: 43–51. 10.1016/j.gene.2004.10.016 15656971

[34] Amberg DC, Burke DJ, Strathern JN. Methods in yeast genetics A Cold Spring Harbor Laboratory Course Manual, Cold Spring Harbor Laboratory Press, Cold Spring Harbor, New York, 2005.

[35] Sherman F. Getting started with yeast. Methods Enzymol. 2002; 350: 3–41. 12073320 10.1016/s0076-6879(02)50954-x

[36] Coen R, Engelberg D. Commonly used *Saccharomyces cerevisiae* strains (e.g. BY4741, W303) are growth sensitive on synthetic complete medium due to poor leucine uptake. FEMS Microbiol Lett. 2007; 273: 239–243. 10.1111/j.1574-6968.2007.00798.x 17573937

[37] Bradford MM. A rapid and sensitive method for the quantitation of microgram quantities of protein utilizing the principle of protein-dye binding. Anal Biochem. 1976; 72: 248–254. 942051 10.1016/0003-2697(76)90527-3

[38] Gillen KM, Pausch M, Dohlman HG. N-terminal domain of Gpa1 (G protein alpha) subunit is sufficient for plasma membrane targeting in yeast *Saccharomyces cerevisiae*. J Cell Sci. 1998; 111 (Pt. 21):3235–3244. 9763517 10.1242/jcs.111.21.3235

[39] Guthrie C., Fink GR (ed.). Guide to yeast genetics and molecular biology. In Metods in Enzymology. 1991; 194: 1–863. 2005781

[40] Palacios O, Atrian S, Capdevila M. Zn- and Cu-thioneins: a functional classification. J Biol Inorg Chem. 2011; 16: 991–1009. 10.1007/s00775-011-0827-2 21823038

[41] Eide DJ. Multiple regulatory mechanisms maintain zinc homeostasis in *Saccharomyces cerevisiae*. J Nutr. 2003; 133: 1532S–1535S. 12730459 10.1093/jn/133.5.1532S

[42] Jin S, Sun D, Wang J, Li Y, Wang X, Liu S. Expression of the rgMT gene, encoding for a rice metallothionein-like protein in *Saccharomyces cerevisiae* and *Arabidopsis thaliana*. J Genet. 2014; 93: 709–18. 25572229 10.1007/s12041-014-0430-8

[43] Tomas M, Pagani MA, Andreo CS, Capdevila M, Atrian S, Bofill R. Sunflower metallothionein family characterisation. Study of the Zn(II)- and Cd(II)-binding abilities of the HaMT1 and HaMT2 isoforms. J Inorg Biochem. 2015; 148: 35–48. 10.1016/j.jinorgbio.2015.02.016 25770010

[44] Geva P, Kahta R, Nakonechny F, Aronov S, Nisnevitch M. Increased copper bioremediation ability of new transgenic and adapted *Saccharomyces cerevisiae* strains. Environ Sci Pollut Res Int. 2016; 23: 19613–19625. 10.1007/s11356-016-7157-4 27392627

[45] Ansarypour Z, Shahpiri A. Heterologous expression of a rice metallothionein isoform (OsMTI-1b) in *Saccharomyces cerevisiae* enhances cadmium, hydrogen peroxide and ethanol tolerance. Braz J Microbiol. 2017; pii: S1517-8382(16)30354-30359. 10.1016/j.bjm.2016.10.024 PMC5498412 28223030

