# Supplementary figures and images for: Anchoring plant metallothioneins to the inner face of the plasma membrane of Saccharomyces cerevisiae cells leads to heavy metal accumulation
Source: PLoS One. 2017 May 31;12(5):e0178393. doi: 10.1371/journal.pone.0178393 (PMC5451056; doi:10.1371/journal.pone.0178393)

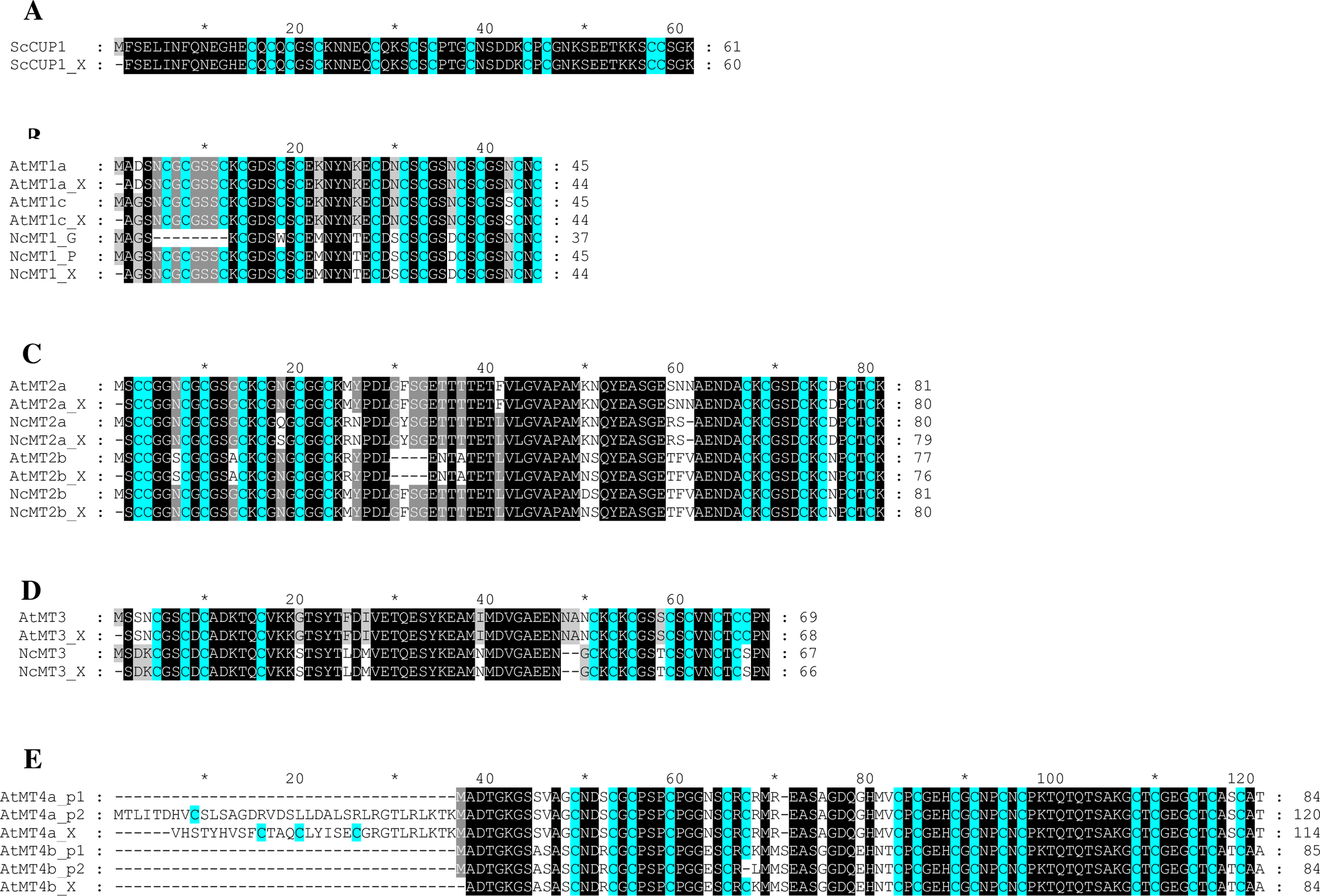

Supplement: S1 Fig — A. ScCUP1 (P0CX80), metallothionein from Saccharomyces cerevisiae. B. Subfamily 1 of Arabidopsis thaliana Col-0 metallothioneins AtMT1a (P43392) and AtMT1c (Q38804) and the Noccaea caerulescens NcMT1 metallothionein from the ecotypes Ganges (NcMT1_G, AAX40656) and Prayon (NcMT1_P, AY486003). Only one NcMT1 ecotype was used to clone NcMT1. C. Subfamily 2 metallothioneins AtMT2a (P25860) and AtMT2b (Q38805) from A. thaliana Col-0 and NcMT2a (ACR46970) and NcMT2b (ACR46961) from N. caerulescens La Calamine. D. Subfamily 3 metallothionein AtMT3 (O22433) from A. thaliana Col-0 and NcMT3 (ACR46965) from N. caerulescens La Calamine. E. The two A. thaliana Col-0 metallothioneins of subfamily 4, which might be present as to polypeptides each (generated by alternative splicing): AtMT4a (p1: P93746, p2: F4ILY7) and AtMT4b (p1: Q42377, p2: F4ILI2). Cysteines are highlighted in blue. (TIF) [file pone.0178393.s001.tif]

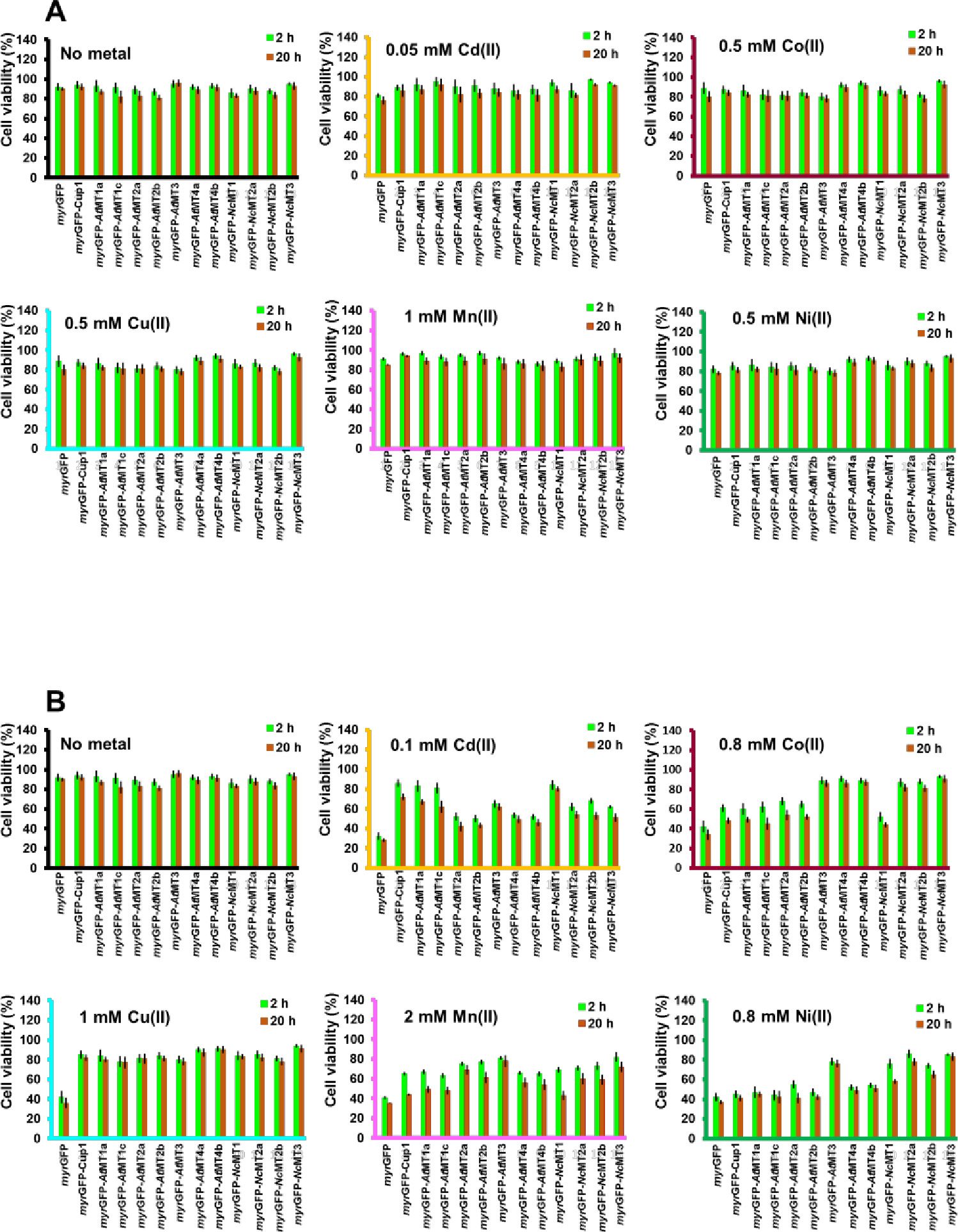

Supplement: S2 Fig — Early log phase growing cells transformed with pGRD-myrGFP::MTx series were shifted to SGal-Ura for transgene induction. Four hours after the galactose shift, MeCl2 was added at the indicated concentration and samples were harvested for viability assay by methylene blue staining. Cell viability was expressed as percentage of live cells within a whole population. Viability was examined for at least 300 cells from one biological replicate. Viable cells were colorless, and dead cells were blue. Values are means ± standard deviation of three independent data. A. Maximum metal concentrations which did not significantly alter cell viability. B. At higher metal concentrations, viability of cells expressing myrGFP-MTx is less affected compared to control myrGFP. (TIF) [file pone.0178393.s002.tif]
