# Supplementary material for: Anchoring plant metallothioneins to the inner face of the plasma membrane of Saccharomyces cerevisiae cells leads to heavy metal accumulation
Source: PLoS One. 2017 May 31;12(5):e0178393. doi: 10.1371/journal.pone.0178393 (PMC5451056; doi:10.1371/journal.pone.0178393)
Supplement: S1 Table — The primers were used to amplify plant MTs cDNAs from Arabidopsis thaliana (At) or Noccaea caerulescens (Nc) cDNAs. The MT gene CUP1 from Saccharomyces cerevisiae (Sc) is intronless and was amplified from genomic DNA. The primers used introduced restriction sites suitable for subsequent subcloning of the amplified fragments into yeast vectors. To amplify the myrGFP control, pGREG596 was used as template. The map of plasmid pGREG596 can be found at (http://web.uni-frankfurt.de/fb15/mikro/euroscarf/data/pGREG.html). (DOCX) [file pone.0178393.s003.docx]

**S1 Table. Primers used to clone plant metallothioneins**

| **Metallothionein** | **Genebank accession number or GeneID** | **Forward primer** | **Reverse primer** | **Insert sequence**  **(fused downstream of *myrGFP)*** |
| --- | --- | --- | --- | --- |
| ***myrGFP***  **(Control)** |  | cgcactagtatggggtgtac | agtcgactcacgaattcttgtatagttcat | - |
|  |  |  |  |  |
| ***Sc*MT (*CUP1*)** | NM_001179185.1 | cggaattcgttcagcgaattaattaacttc | atgtcgactcatttcccagagcagcatgac | aattcgttcagcgaattaattaacttcCAAAATGAAGGTCATGAGTGCCAATGCCAATGTGGTAGCTGCAAAAATAATGAACAATGCCAAAAATCATGTAGCTGCCCAACGGGGTGTAACAGCGACGACAAATGCCCCTGCGGTAACAAGTCTGAAGAAACCAAGAAgtcatgctgctctgggaaatgagtcga |
| ***At*MT1a** | AT1G07600.1 | aggaattcggcagattctaac | tagtcgactcaacagttacagtttg | aattcggcagattctaacTGTGGATGTGGCTCCTCCTGCAAATGTGGTGACTCTTGCA  GTTGCGAGAAGAACTACAACAAGGAGTGCGACAACTGTAGCTGTGGATCAAACTGCAGCTGTGGGTcaaactgtaactgttgagtcga |
| ***At*MT1c** | AT1G07610.1 | gaagtaaggaattcggcagg | gatagtcgactcaacagttacagc | aattcggcaggTTCTAACTGTGGATGTGGCTCCTCCTGCAAATGTGGTGATTCGTGCAGTTGCGAGAAGAACTACAACAAGGAGTGTGATAACTGTAGCTGTGGATCAAACTGCAGCTGCGGGTCAAgctgtaactgttgagtcga |
| ***At*MT2a** | AT3G09390.1 | aggaattcgtcttgctgtgga | aggtcgactcacttgcaggtg | aattcgtcttgctgtggaGGAAACTGCGGATGTGGATCTGGCTGCAAGTGCGGCAAC  GGTTGTGGAGGTTGCAAAATGTACCCTGACTTGGGATTCTCCGGCGAGACAACCACAACTGAGACTTTTGTCTTGGGCGTTGCACCGGCGATGAAGAATCAGTACGAGGCTTCAGGGGAGAGTAACAACGCTGAGAACGATGCTTGCAAGTGTGGATCTGACTGCAAGTGTGATCCTTGcacctgcaagtgagtcga |
| ***At*MT2b** | AT5G02380.1 | aggaattcgtcttgctgtgg | ctgtcgactcatttgcaggta | aattcgtcttgctgtggTGGAAGCTGTGGTTGTGGATCTGCCTGCAAGTGCGGCAATGGTTGCGGAGGTTGCAAAAGGTACCCTGACTTGGAGAACACCGCCACCGAGACTCTTGTCCTCGGTGTTGCTCCGGCGATGAACTCTCAGTACGAGGCTTCCGGCGAGACTTTCGTTGCCGAGAATGATGCTTGCAAATGCGGATCTGACTGCAAGTGCAACCCTTGtacctgcaaatgagtcga |
| ***At*MT3** | AT3G15353.1 | tcgaattcgtcaagcaactg | aagtcgacttagttggggcag | aattcgtcaagcaactgCGGAAGCTGCGACTGTGCTGACAAGACCCAGTGCGTAAAGAAGGGAACCAGCTACACCTTCGACATCGTCGAGACTCAGGAGAGCTACAAGGAGGCCATGATCATGGACGTTGGTGCCGAGGAGAACAACGCAAATTGCAAGTGCAAGTGCGGCTCCTCTTGCAGCTGCGTCAACTGCACTTGctgccccaactaagtcga |
| ***At*MT4a** | AT2G42000.1 | agggatccgtacatagtacttaccacg | gagctcgagatgtctaagtgg | gatccgtacatagtacttaccacgTATCCTTTTGCACTGCCCAGTGTCTATATATATCTGAATGTGGCAGAGGGACTTTAAGATTGAAAACGAAAATGGCAGATACAGGCAAAGGAAGCTCTGTCGCTGGCTGCAACGATAGCTGTGGCTGCCCTTCTCCCTGTCCCGGTGGGAATTCCTGCAGGTGCAGGATGAGAGAAGCATCTGCTGGGGATCAAGGGCACATGGTGTGCCCGTGTGGGGAGCACTGCGGATGCAACCCCTGCAACTGCCCCAAGACCCAAACCCAAACCTCCGCCAAGGGCTGCACCTGTGGTGAGGGTTGCACATGCGCCTCTTGCGccacttagacatctcga |
| ***At*MT4b** | AT2G23240.1 | acagaattcggcagacacag | catagtcgacgatgtctaagca | aattcggcagacacagGCAAAGGAAGTGCAAGCGCTAGCTGCAACGATCGTTGTGGCTGCCCTTCTCCATGTCCCGGTGGCGAATCTTGCAGGTGCAAGATGATGAGCGAAGCATCTGGTGGGGATCAAGAGCACAACACGTGTCCATGTGGGGAGCACTGTGGCTGTAACCCTTGCAACTGCCCCAAGACTCAAACTCAAACCTCTGCTAAGGGTTGCACTTGTGGTGAGGGCTGCACCTGTGCCACTTGCGCtgcttagacatcgtcga |
| ***Nc*MT1** | AY847454.1 | gagaattcggccggttctaa | ccacgtcgactcaacagttg | aattcggccggttctaaCTGTGGATGTGGTTCCTCCTGCAAATGTGGTGACTCTTGCAGTTGCGAGATGAACTACAACACGGAGTGCGACAGCTGCAGCTGTGGATCAGACTGCAGCTGTGGGTCAAACTGcaactgttgagtcga |
| ***Nc*MT2a** | FJ439656.1  FJ439654.1 | aggaattcgtcttgctgcg | ggtttatgtcgactcatttacaggtg | aattcgtcttgctgcgGAGGAAACTGTGGTTGTGGATCTGGCTGCAAGTGCGGCAGCGGTTGCGGAGGTTGCAAAAGGAACCCAGACTTGGGATATTCCGGGGAGACGACCACGACCGAGACACTTGTCTTGGGCGTTGCACCGGCGATGAAGAACCAGTACGAGGCTTCCGGCGAGAGGAGCGCTGAGAACGATGCTTGCAAGTGTGGATCTGACTGCAAGTGCGACCCTTGcacctgtaaatgagtcga |
| ***Nc*MT2b** | FJ439648.1  FJ439647.1 | aggaattcgtcttgctgtgg | ctgtcgactcatttgcaggta | aattcgtcttgctgtggAGGAAACTGTGGTTGCGGATCTGGCTGCAAGTGCGGCAACGGATGCGGAGGTTGCAAAATGTACCCAGACTTGGGTTTCTCTGGTGAGACCACCACCACCGAGACTCTTGTCCTCGGCGTTGCCCCGGCGATGAACTCCCAGTACGAGGCTTCCGGCGAGACCTTCGTTGCCGAGAATGATGCCTGCAAATGCGGATCTGACTGCAAGTGCAACCCTTGtacctgcaaatgagtcga |
| ***Nc*MT3** | FJ439655.1  FJ439651.1 | ccgaattcgtcggacaagtg | ctgtcgacttagttggggctg | aattcgtcggacaagtgCGGAAGCTGCGACTGTGCTGACAAGACCCAGTGCGTCAAGAAGAGTACCAGCTACACCTTGGACATGGTCGAGACTCAGGAGAGCTACAAGGAGGCCATGAACATGGACGTTGGTGCAGAAGAGAACGGGTGCAAATGCAAGTGCGGCTCTACCTGCAGCTGCGTCAACTGCACTTGcagccccaactaagtcga |

The primers were used to amplify the complete coding sequence of metallothioneins from *Arabidopsis thaliana* (*At*) or *Noccaea caerulescens* (*Nc*) cDNA. The metallothionein gene *CUP1* from *Saccharomyces cerevisiae* (*Sc*) is intronless and was amplified from genomic DNA. The primers introduced restriction sites suitable for subsequent subcloning of the amplified fragments into yeast vectors. To amplify the *myr*GFP control, pGREG596 (http://web.uni-frankfurt.de/fb15/mikro/euroscarf/data/pGREG.html) was used as template.

Lower cases: primer sequences. Highlighted text: restriction sites. *Spe*I site; *EcoR*I site; *Sal*I site; *Bam*HI site; *Xho*I site; STOP codon.
