# Supplementary material for: Anchoring plant metallothioneins to the inner face of the plasma membrane of Saccharomyces cerevisiae cells leads to heavy metal accumulation
Source: PLoS One. 2017 May 31;12(5):e0178393. doi: 10.1371/journal.pone.0178393 (PMC5451056; doi:10.1371/journal.pone.0178393)
Supplement: S2 Table — (DOCX) [file pone.0178393.s004.docx]

**S2 Table. Amino acid sequence of the chimeric metallothioneins (*myr*GFP-MTx) expressed in yeast cells**

| **Protein expressed** | **Sequence** |
| --- | --- |
| ***myr*GFP (control)** | MGCTVSTQTISKGEELFTGVVPILVELDGDVNGHKFSVSGEGEGDATYGKLTLKFICTTGKLPVPWPTLVTTFTYGVQCFSRYPDHMKQHDFFKSAMPEGYVQERTIFFKDDGNYKTRAEVKFEGDTLVNRIELKGIDFKEDGNILGHKLEYNYNSHNVYIMADKQKNGIKVNFKIRHNIEDGSVQLADHYQQNTPIGDGPVLLPDNHYLSTQSALSKDPNEKRDHMVLLEFVTAAGITHGMDELYKNS* |
| ***myr*GFP*-*Cup1** | MGCTVSTQTISKGEELFTGVVPILVELDGDVNGHKFSVSGEGEGDATYGKLTLKFICTTGKLPVPWPTLVTTFTYGVQCFSRYPDHMKQHDFFKSAMPEGYVQERTIFFKDDGNYKTRAEVKFEGDTLVNRIELKGIDFKEDGNILGHKLEYNYNSHNVYIMADKQKNGIKVNFKIRHNIEDGSVQLADHYQQNTPIGDGPVLLPDNHYLSTQSALSKDPNEKRDHMVLLEFVTAAGITHGMDELYKNSFSELINFQNEGHECQCQCGSCKNNEQCQKSCSCPTGCNSDDKCPCGNKSEETKKSCCSGK* |
| ***myr*GFP-*At*MT1a** | MGCTVSTQTISKGEELFTGVVPILVELDGDVNGHKFSVSGEGEGDATYGKLTLKFICTTGKLPVPWPTLVTTFTYGVQCFSRYPDHMKQHDFFKSAMPEGYVQERTIFFKDDGNYKTRAEVKFEGDTLVNRIELKGIDFKEDGNILGHKLEYNYNSHNVYIMADKQKNGIKVNFKIRHNIEDGSVQLADHYQQNTPIGDGPVLLPDNHYLSTQSALSKDPNEKRDHMVLLEFVTAAGITHGMDELYKNSADSNCGCGSSCKCGDSCSCEKNYNKECDNCSCGSNCSCGSNCNC* |
| ***myr*GFP-*At*MT1c** | MGCTVSTQTISKGEELFTGVVPILVELDGDVNGHKFSVSGEGEGDATYGKLTLKFICTTGKLPVPWPTLVTTFTYGVQCFSRYPDHMKQHDFFKSAMPEGYVQERTIFFKDDGNYKTRAEVKFEGDTLVNRIELKGIDFKEDGNILGHKLEYNYNSHNVYIMADKQKNGIKVNFKIRHNIEDGSVQLADHYQQNTPIGDGPVLLPDNHYLSTQSALSKDPNEKRDHMVLLEFVTAAGITHGMDELYKNSAGSNCGCGSSCKCGDSCSCEKNYNKECDNCSCGSNCSCGSSCNC* |
| ***myr*GFP-*At*MT2a** | MGCTVSTQTISKGEELFTGVVPILVELDGDVNGHKFSVSGEGEGDATYGKLTLKFICTTGKLPVPWPTLVTTFTYGVQCFSRYPDHMKQHDFFKSAMPEGYVQERTIFFKDDGNYKTRAEVKFEGDTLVNRIELKGIDFKEDGNILGHKLEYNYNSHNVYIMADKQKNGIKVNFKIRHNIEDGSVQLADHYQQNTPIGDGPVLLPDNHYLSTQSALSKDPNEKRDHMVLLEFVTAAGITHGMDELYKNSSCCGGNCGCGSGCKCGNGCGGCKMYPDLGFSGETTTTETFVLGVAPAMKNQYEASGESNNAENDACKCGSDCKCDPCTCK* |
| ***myr*GFP-*At*MT2b** | MGCTVSTQTISKGEELFTGVVPILVELDGDVNGHKFSVSGEGEGDATYGKLTLKFICTTGKLPVPWPTLVTTFTYGVQCFSRYPDHMKQHDFFKSAMPEGYVQERTIFFKDDGNYKTRAEVKFEGDTLVNRIELKGIDFKEDGNILGHKLEYNYNSHNVYIMADKQKNGIKVNFKIRHNIEDGSVQLADHYQQNTPIGDGPVLLPDNHYLSTQSALSKDPNEKRDHMVLLEFVTAAGITHGMDELYKNSSCCGGSCGCGSACKCGNGCGGCKRYPDLENTATETLVLGVAPAMNSQYEASGETFVAENDACKCGSDCKCNPCTCK* |
| ***myr*GFP-*At*MT3** | MGCTVSTQTISKGEELFTGVVPILVELDGDVNGHKFSVSGEGEGDATYGKLTLKFICTTGKLPVPWPTLVTTFTYGVQCFSRYPDHMKQHDFFKSAMPEGYVQERTIFFKDDGNYKTRAEVKFEGDTLVNRIELKGIDFKEDGNILGHKLEYNYNSHNVYIMADKQKNGIKVNFKIRHNIEDGSVQLADHYQQNTPIGDGPVLLPDNHYLSTQSALSKDPNEKRDHMVLLEFVTAAGITHGMDELYKNSSSNCGSCDCADKTQCVKKGTSYTFDIVETQESYKEAMIMDVGAEENNANCKCKCGSSCSCVNCTCCPN* |
| ***myr*GFP-*At*MT4a** | MGCTVSTQTISKGEELFTGVVPILVELDGDVNGHKFSVSGEGEGDATYGKLTLKFICTTGKLPVPWPTLVTTFTYGVQCFSRYPDHMKQHDFFKSAMPEGYVQERTIFFKDDGNYKTRAEVKFEGDTLVNRIELKGIDFKEDGNILGHKLEYNYNSHNVYIMADKQKNGIKVNFKIRHNIEDGSVQLADHYQQNTPIGDGPVLLPDNHYLSTQSALSKDPNEKRDHMVLLEFVTAAGITHGMDELYKNS ISSLSIPSTSYGSVHSTYHVSFCTAQCLYISECGRGTLRLKTKMADTGKGSS VAGCNDSCGCPSPCPGGNSCRCRMREASAGDQGHMVCPCGEHCGCNPCNCPKTQTQTSAKGCTCGEGCTCASCAT* |
| ***myr*GFP-*At*MT4b** | MGCTVSTQTISKGEELFTGVVPILVELDGDVNGHKFSVSGEGEGDATYGKLTLKFICTTGKLPVPWPTLVTTFTYGVQCFSRYPDHMKQHDFFKSAMPEGYVQERTIFFKDDGNYKTRAEVKFEGDTLVNRIELKGIDFKEDGNILGHKLEYNYNSHNVYIMADKQKNGIKVNFKIRHNIEDGSVQLADHYQQNTPIGDGPVLLPDNHYLSTQSALSKDPNEKRDHMVLLEFVTAAGITHGMDELYKNSADTGKGSASASCNDRCGCPSPCPGGESCRCKMMSEASGGDQEHNTCPCGEHCGCNPCNCPKTQTQTSAKGCTCGEGCTCATCAA* |
| ***myr*GFP-*Nc*MT1** | MGCTVSTQTISKGEELFTGVVPILVELDGDVNGHKFSVSGEGEGDATYGKLTLKFICTTGKLPVPWPTLVTTFTYGVQCFSRYPDHMKQHDFFKSAMPEGYVQERTIFFKDDGNYKTRAEVKFEGDTLVNRIELKGIDFKEDGNILGHKLEYNYNSHNVYIMADKQKNGIKVNFKIRHNIEDGSVQLADHYQQNTPIGDGPVLLPDNHYLSTQSALSKDPNEKRDHMVLLEFVTAAGITHGMDELYKNSSAGSNCGCGSSCKCGDSCSCEMNYNTECDSCSCGSDCSCGSNCNC* |
| ***myr*GFP-*Nc*MT2a** | MGCTVSTQTISKGEELFTGVVPILVELDGDVNGHKFSVSGEGEGDATYGKLTLKFICTTGKLPVPWPTLVTTFTYGVQCFSRYPDHMKQHDFFKSAMPEGYVQERTIFFKDDGNYKTRAEVKFEGDTLVNRIELKGIDFKEDGNILGHKLEYNYNSHNVYIMADKQKNGIKVNFKIRHNIEDGSVQLADHYQQNTPIGDGPVLLPDNHYLSTQSALSKDPNEKRDHMVLLEFVTAAGITHGMDELYKNSSCCGGNCGCGSGCKCGSGCGGCKRNPDLGYSGETTTTETLVLGVAPAMKNQYEASGERSAENDACKCGSDCKCDPCTCK* |
| ***myr*GFP-*Nc*MT2b** | MGCTVSTQTISKGEELFTGVVPILVELDGDVNGHKFSVSGEGEGDATYGKLTLKFICTTGKLPVPWPTLVTTFTYGVQCFSRYPDHMKQHDFFKSAMPEGYVQERTIFFKDDGNYKTRAEVKFEGDTLVNRIELKGIDFKEDGNILGHKLEYNYNSHNVYIMADKQKNGIKVNFKIRHNIEDGSVQLADHYQQNTPIGDGPVLLPDNHYLSTQSALSKDPNEKRDHMVLLEFVTAAGITHGMDELYKNSSCCGGNCGCGSGCKCGNGCGGCKMYPDLGFSGETTTTETLVLGVAPAMNSQYEASGETFVAENDACKCGSDCKCNPCTCK* |
| ***myr*GFP-*Nc*MT3** | MGCTVSTQTISKGEELFTGVVPILVELDGDVNGHKFSVSGEGEGDATYGKLTLKFICTTGKLPVPWPTLVTTFTYGVQCFSRYPDHMKQHDFFKSAMPEGYVQERTIFFKDDGNYKTRAEVKFEGDTLVNRIELKGIDFKEDGNILGHKLEYNYNSHNVYIMADKQKNGIKVNFKIRHNIEDGSVQLADHYQQNTPIGDGPVLLPDNHYLSTQSALSKDPNEKRDHMVLLEFVTAAGITHGMDELYKNSSDKCGSCDCADKTQCVKKSTSYTLDMVETQESYKEAMNMDVGAEENGCKCKCGSTCSCVNCTCSPN* |

Myristoylation sequence. GFP sequence. Linker sequence. Metallothionein sequence. *STOP
