# Supplementary material for: Anchoring plant metallothioneins to the inner face of the plasma membrane of Saccharomyces cerevisiae cells leads to heavy metal accumulation
Source: PLoS One. 2017 May 31;12(5):e0178393. doi: 10.1371/journal.pone.0178393 (PMC5451056; doi:10.1371/journal.pone.0178393)
Supplement: S3 Table — The forward primer (universal) overlapped nucleotides 8–27 of myrGFP fragment, while the reverse primer complemented the fragment 48–67 of each fused MT cDNA. (DOCX) [file pone.0178393.s005.docx]

**S3 Table. Primers used to assess the transcript level of *myrGFP::MT* transgenes in yeast cells by Reverse Transcription-PCR (RT-PCR)**

| **Metallothionein cDNA** | **Forward primer (universal)** | **Reverse primer (specific)** |
| --- | --- | --- |
| ***myrGFPΔ*** | GTACAGTGAGTACGCAAACA | TCGACGGTATCGATAAGCTT |
| ***Sc*MT (*CUP1*)** |  | CAGCTACCACATTGGCATTG |
| ***At*MT1a** |  | GTTGTAGTTCTTCTCGCAAC |
| ***At*MT1c** |  | TCTCGCAACTGCACGAATCA |
| ***At*MT2a** |  | TCCACAACCGTTGCCGCACT |
| ***At*MT2b** |  | TCCGCAACCATTGCCGCACT |
| ***At*MT3** |  | TTCCCTTCTTTACGCACTGG |
| ***At*MT4a** |  | TCTGCCACATTCAGATATAT |
| ***At*MT4b** |  | AAGGGCAGCCACAACGATCG |
| ***Nc*MT1** |  | ATCTCGCAACTGCAAGAGTC |
| ***Nc*MT2a** |  | TCCGCAACCGCTGCCGCACT |
| ***Nc*MT2b** |  | TCCGCATCCGTTGCCGCACT |
| ***Nc*MT3** |  | TGGTACTCTTCTTGACGCAC |
|  | | |
| ***ACT1* (control)** | GGTTGCTGCTTTGGTTATTG | CAATTGGGTAACGTAAAGTC |

The forward primer (universal) overlapped nucleotides 8-27 of the myrGFP fragment, while the reverse primer annealed to the position 48-67 of each fused MT cDNA.
